# Biomimetic Self‐Reconfigurable Soft Gripper for Cross‐Scale, Multi‐Particle, and High‐Load Multifunctional Manipulation

**DOI:** 10.1002/advs.76403

**Published:** 2026-07-03

**Authors:** Qiping Xu, Bin Wang, Jinxin Chen, Zhengqiang Guo, Baisong Yang, Chaoqian Chen, Jiancheng Cai, Chee‐Meng Chew, Shiju E

**Affiliations:** ^1^ Key Laboratory of Urban Rail Transit Intelligent Operation and Maintenance Technology and Equipment of Zhejiang Province Department of Robotics Engineering College of Engineering Zhejiang Normal University Jinhua P. R. China; ^2^ Department of Mechanical Engineering College of Design and Engineering 9 Engineering Drive 1 National University of Singapore Singapore Singapore

**Keywords:** altering workspace, bioinspired, highly integrated, monolithically 3D‐printed, self‐reconfigurable, soft gripper, switching finger arrangements

## Abstract

Soft robotic grippers, with their intrinsic compliance and dexterity, provide safer manipulation of soft and fragile items compared to traditional rigid ones. However, achieving high functional integration within a single soft gripper, particularly for cross‐scale, multi‐particle, and high‐load manipulation, remains a major challenge. Here, a monolithically 3D‐printed, rapeseed‐flower‐inspired self‐reconfigurable soft gripper (SRSG) is presented, which can rapidly reconfigure its finger arrangement within ∼130 ms and achieves precise, reversible switching between diagonal and parallel configurations. The SRSG can be readily incorporated with detachable petal modules to alter the grasping workspace. Leveraging these capabilities enables a range of functions: rotating bulbs of varying diameters, picking fruits, grasping cross‐scale objects ranging from 0.07 to 270 mm (grasping range ratio of ∼3857 times), lifting payloads up to 5.6 kg (∼106 times its own weight), and adaptively enveloping numerous fine particles, multiple live aquatic organisms, and fragile underwater targets. The fully soft, electronics‐free SRSG establishes a self‐reconfigurable grasping paradigm for robust operation in unstructured environments, and opens up new directions for soft robotic end‐effectors.

## Graphical abstract

A fully soft, self‐reconfigurable gripper inspired by rapeseed flowers is monolithically 3D‐printed and electronics‐free, switching between diagonal and parallel finger arrangements. Establishing a self‐reconfigurable grasping paradigm, the gripper enables diverse manipulation tasks, including rotating bulbs, picking fruits, grasping cross‐scale objects, lifting large loads, enveloping scattered particles, live aquatic organisms, and fragile underwater targets.

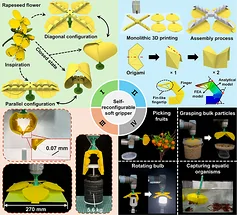

## 1 Introduction

Animals and plants in nature dynamically reconfigure their morphology in response to environmental changes [1, 2]. Inspired by these biological strategies [3, 4], numerous soft robotic grippers have been developed to replicate compliant deformation and flexible grasping similar to their natural counterparts [5]. Compared with conventional rigid grippers, soft grippers are inherently safer for interaction in human‐centered and unstructured environments, thanks to their intrinsic compliance [6]. As a result, they have found widespread applications in areas ranging from industrial sorting and agricultural harvesting to deep‐sea exploration and minimally invasive surgery. Consequently, soft grippers are emerging as essential tools for safe, adaptive, and dexterous manipulation across diverse real‐world settings [7, 8, 9, 10, 11].

To meet diverse application demands, a broad range of soft grippers with distinct architectures has been developed, including finger‐type, suction‐type, continuum‐type, foldable‐type, and wrapping‐type designs [12, 13, 14, 15, 16]. Among these, finger‐type soft grippers are the most widely adopted owing to their simple construction, high reproducibility, and broad versatility [17]. However, most finger‐type grippers are built with fixed finger layouts [18, 19, 20], which fundamentally limit their ability to manipulate irregular and heterogeneous objects while significantly compromising their cross‐scale and high‐load capabilities. For instance, concentric or parallel staggered finger arrangements constrain fingertip contact, making it difficult to grasp small objects, whereas parallel symmetrical finger arrangements lack geometric closure when gripping circular cross‐section objects, resulting in frequent slippage and unstable grasping [21, 22, 23]. These limitations underscore that fixed finger arrangements restrict the gripper's adaptability, undermining both grasping stability and operational versatility. To address this reconfiguration challenge, current research primarily employs two strategies: (i) manually replacing fingers of different shapes and sizes along with their corresponding rigid bases [24, 25, 26], and (ii) integrating rigid transmission mechanisms to reposition finger bases and thereby alter finger arrangements [27, 28, 29, 30]. Nevertheless, the former sacrifices operational continuity and productivity, while the latter introduces structural redundancy, raises manufacturing costs, and increases assembly and control complexity. As such, developing a structurally simple, fully soft, and self‐reconfigurable monolithic soft gripper capable of rapidly switching finger arrangements remains a key unsolved challenge.

Although finger‐type grippers are straightforward to operate and control, their discrete finger layout makes them difficult to grasp granular items much smaller than the finger cross‐section or capture multiple small live organisms. As a result, their performance remains constrained when dealing with multi‐particle manipulation and bulk enveloping tasks, forming a persistent technical bottleneck. Proposed remedies include adding disc‐shaped ventral membranes between fingers to fill grasping gaps [31, 32], inserting silicone sheets between fingers to improve enveloping capability [33, 34], or widening fingers to wrap multiple objects at once [35, 36]. However, these strategies suffer from two inherent drawbacks: (i) noticeable gaps often remain even when the gripper is fully closed, preventing a sealed enclosure and allowing fine particles or small organisms to escape during manipulation; and (ii) the added modules are permanently attached, restricting the gripper's adaptive deformation, compromising both dexterity and output force. Consequently, there is a pressing need for a detachable module that can form a sealed grasping volume when needed, yet be removed when unnecessary, enabling reconfiguration of the gripper's workspace while avoiding structural redundancy and performance degradation.

Here, we introduce a rapeseed‐flower‐inspired self‐reconfigurable soft gripper (SRSG) that can rapidly switch between diagonal and parallel finger layouts while reversibly transitioning between fully unfolded and grasping closed states, thereby establishing a self‐reconfigurable grasping paradigm featuring switchable finger arrangements and adjustable grasping workspaces. The SRSG is entirely soft and electronics‐free, and monolithically fabricated via fused deposition modeling (FDM) 3D printing. The SRSG enables self‐reconfiguration by U‐shaped origami chambers embedded within the finger and palm joints, which produce reversible and fast configuration transformation without compromising compliance under simple pneumatic actuation. To broaden manipulation capability, a fin‐like fingertip design is incorporated into the gripper to support adaptive pinching and conforming contact. Systematic characterization confirms that an appropriate gripper configuration significantly improves both output force and grasping success rate. We validate multifunctional manipulation in representative tasks, including rotating light bulbs (70 to 135 mm diameter), enveloping bulk rice grains (∼1 mm), capturing multiple live aquatic organisms with stress response and complex morphologies, handling fragile porcelain items underwater, and grasping irregular fruits, vegetables, and everyday objects spanning three orders of magnitude in size (0.07 to 270 mm). These demonstrations collectively highlight the SRSG's potential as a highly integrated, high‐performance, and versatile soft robotic end‐effector.

## 2 Results

### 2.1 Design, Fabrication, and Operation Principles of the SRSG

We drew inspiration from natural rapeseed flowers, observing that when the rapeseed flowers close, their petals overlap to form a nearly enclosed structure that protects the pistil. When opened, the four petals unfold into a fully flattened configuration, arranged either diagonally or in nearly parallel pairs on opposite sides of the receptacle (Figure 1A, gray dashed circle) [37]. Compared with other flowers that exhibit only opening and closing behaviors, the rapeseed flower possesses several biological morphological features directly related to the gripper design. Inspired by this, we designed a highly integrated SRSG (Figure 1A). The nearly planar open state and symmetric petal morphology provide a biological analogue for the coplanar initial layout of the SRSG's four fingers and central palm, thereby helping to expand its accessible grasping workspace. The diagonal or nearly parallel petal arrangements inspired two types of switchable finger configurations of the SRSG. In addition, the orderly staggered overlapping petal‐enclosure mechanism motivated the development of detachable “petal” modules (hereafter referred to as petals) for enveloping grasping. To reproduce this mechanism while avoiding mutual interference between adjacent modules, the detachable petals were mounted at different vertical heights. In the unactuated state, the gripper's four fingers are arranged diagonally, while applying negative pressure to the four joints in the palm induces joint rotation, causing the fingers to switch to a parallel arrangement. Similarly, the finger‐bending deformation can be adjusted by modulating negative pressure. When four fingers are actuated, the gripper equipped with four detachable petals transforms into a nearly enclosed configuration capable of enveloping target objects (Movie S1). The design and closed‐state simulations of the petal are provided in Figures S1A, S2, and Text S1. To enable rapid, reversible joint deformation, we designed U‐shaped origami chambers based on origami principles and embedded them into both the fingers and palm joints [38]. These chambers generate controllable folding deformations, substantially reducing mechanical complexity while markedly increasing response speed, enabling the SRSG to complete self‐reconfiguration in roughly 130 ms. The SRSG contains no rigid or electronic components, preserving full compliance and avoiding structural redundancy inherent to hybrid rigid‐soft designs, while unifying structural simplicity with rapid, self‐adaptive reconfiguration. These characteristics enable the SRSG to achieve cross‐scale, multi‐particle, and high‐load grasping, demonstrating a high degree of functional integration and grasping flexibility.

**FIGURE 1:**
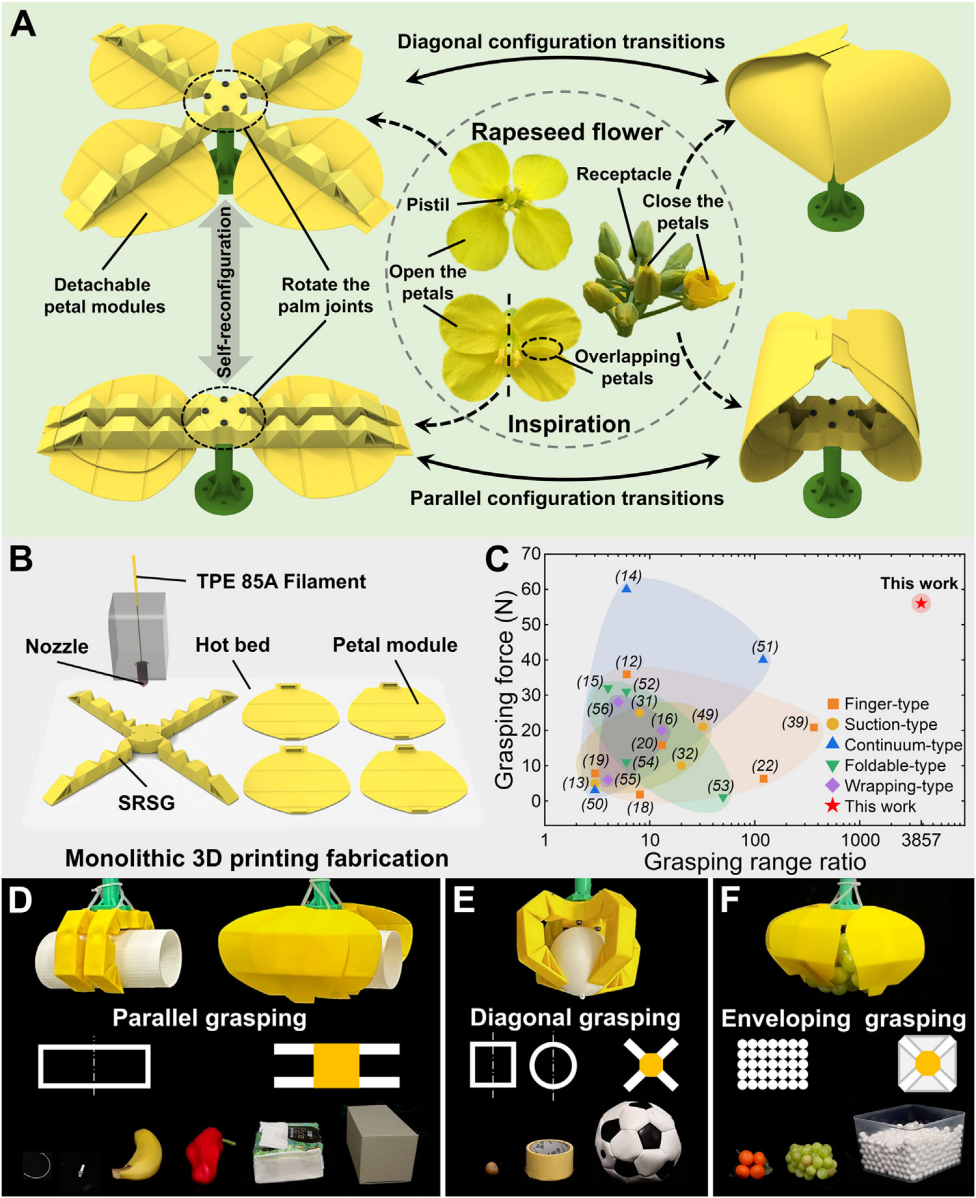
Bioinspired design, fabrication, and applications of the SRSG. (A) Inspired by the petal arrangements and closure mechanism of the rapeseed flower, we proposed a SRSG that can reconfigure its finger layout to switch between diagonal and parallel configurations. (B) Design and fabrication of the monolithic SRSG and four detachable petals, both of which are fabricated by FDM 3D printing. (C) Comparison of the proposed SRSG with representative soft grippers (including finger‐type [12, 18, 19, 20, 22, 39], suction‐type [13, 31, 32, 49], continuum‐type [14, 50, 51], foldable‐type [15, 52, 53, 54], and wrapping‐type [16, 55, 56] grippers) in terms of grasping range ratio (defined as the ratio of the maximum to minimum grasping size) and grasping force. (D−F) The SRSG operates in three distinct grasping modes: (D) parallel grasping mode, the fingers arranged in parallel (with or without petals); (E) diagonal grasping mode, the fingers arranged diagonally (without petals); (F) enveloping grasping mode, the fingers arranged diagonally (with petals).

All components of the SRSG are fabricated from a thermoplastic elastomer (TPE 85A filament) using FDM 3D printing (Figure 1B, Figure S3, and Movie S2). Compared with conventional approaches such as fabric sewing and silicone injection molding [39, 40], FDM printing provides a faster, more convenient, and lower‐cost fabrication solution (the entire SRSG costs only US$0.9). Notably, the SRSG monolithically integrates four fingers and the central palm into a single unit, eliminating the need for labor‐intensive assembly, repeated finger reattachments, or external transmission mechanisms common in previously reported reconfigurable grippers. This monolithic design delivers superior overall performance to existing devices in both load‐bearing capacity and grasping range (Figure 1C). The integration of the detachable petals is mainly intended to complement and extend the functionality of the SRSG in specific grasping scenarios, particularly for enveloping grasping of multiple scattered objects. Accordingly, the gripper can switch between three distinct grasping modes (i.e., parallel, diagonal, and enveloping modes) without cumbersome manual assembly and disassembly of fingers and the base. In the present design, the only manual operation required is the rapid attachment or removal of the optional petals. The SRSG assembly and petal‐mounting procedures are detailed in Figure S1B and Movie S3. The pneumatic circuit schematic of the SRSG is provided in Figure S1C.

The SRSG features parallel, diagonal, and enveloping grasping modes (Figure 1D–F), enabling dexterous manipulation of objects with diverse shapes, sizes, weights, and quantities in unstructured environments. The parallel grasping mode, with or without petals, is well suited for elongated objects (Figure 1D). The diagonal grasping mode without petals is appropriate for objects with square or circular cross‐sections (Figure 1E). The enveloping grasping mode with petals is suited to simultaneously picking up large numbers of scattered objects of varying sizes (Figure 1F). By assessing the object's shape and quantity before operation, the appropriate mode can be rapidly selected, enabling adaptive grasping.

### 2.2 Structural Design, Simulations, and Experiments of the Fingers and Palm

To evaluate the deformation behavior of the SRSG, we examined the bending performance of four fingers (Fingers A−D) with origami chambers (Movie S4) and the central palm joints, as well as the folding process of the U‐shaped origami chamber. Given that the base segment *L*_0_ of each finger is fixed, the bending deformation is governed by three parameters: the joint rotation angle *θ_i_*, the number of segments *N*, and the segment length *L_i_* (see the analytical model in the simulation section of Figure 2A). Analogous to human three‐segment fingers (excluding the thumb), the designed fingers consist of three segments of unequal length, and their deformation depends primarily on the joint rotation angles. We established a kinematic model based on the Denavit–Hartenberg (D–H) parametric method (see Text S2, Figure S4, and Tables S1 and S2) to predict the tip trajectory and bending deformation of the fingers. Subsequently, we conducted finite element analysis (FEA) and experimental validation under a negative pressure of 98 kPa (details in Experimental Section). The material parameters of hyperelastic constitutive models are provided in Text S3 and Figure S5. As shown in Figure 2A–D, the results from FEA, theoretical predictions, and experiments are in good agreement (more comparisons are provided in Figures S6 and S7). Both the theoretical and finite element models reliably capture the finger bending behavior and tip trajectory, providing a robust basis for structural optimization. The origami chamber structures of four different fingers were compared to analyze the influence of the petal, effective chamber volume, and surface creases on finger bending deformation. Finger A and Finger B share the same U‐shaped origami chamber joint, with Finger B incorporating a detachable petal. Finger C has a V‐shaped origami chamber joint with a smaller effective chamber volume, but retains the same surface creases as Finger A. Finger D features the same U‐shaped chamber profile as Finger A, but contains crease‐free surfaces.

**FIGURE 2:**
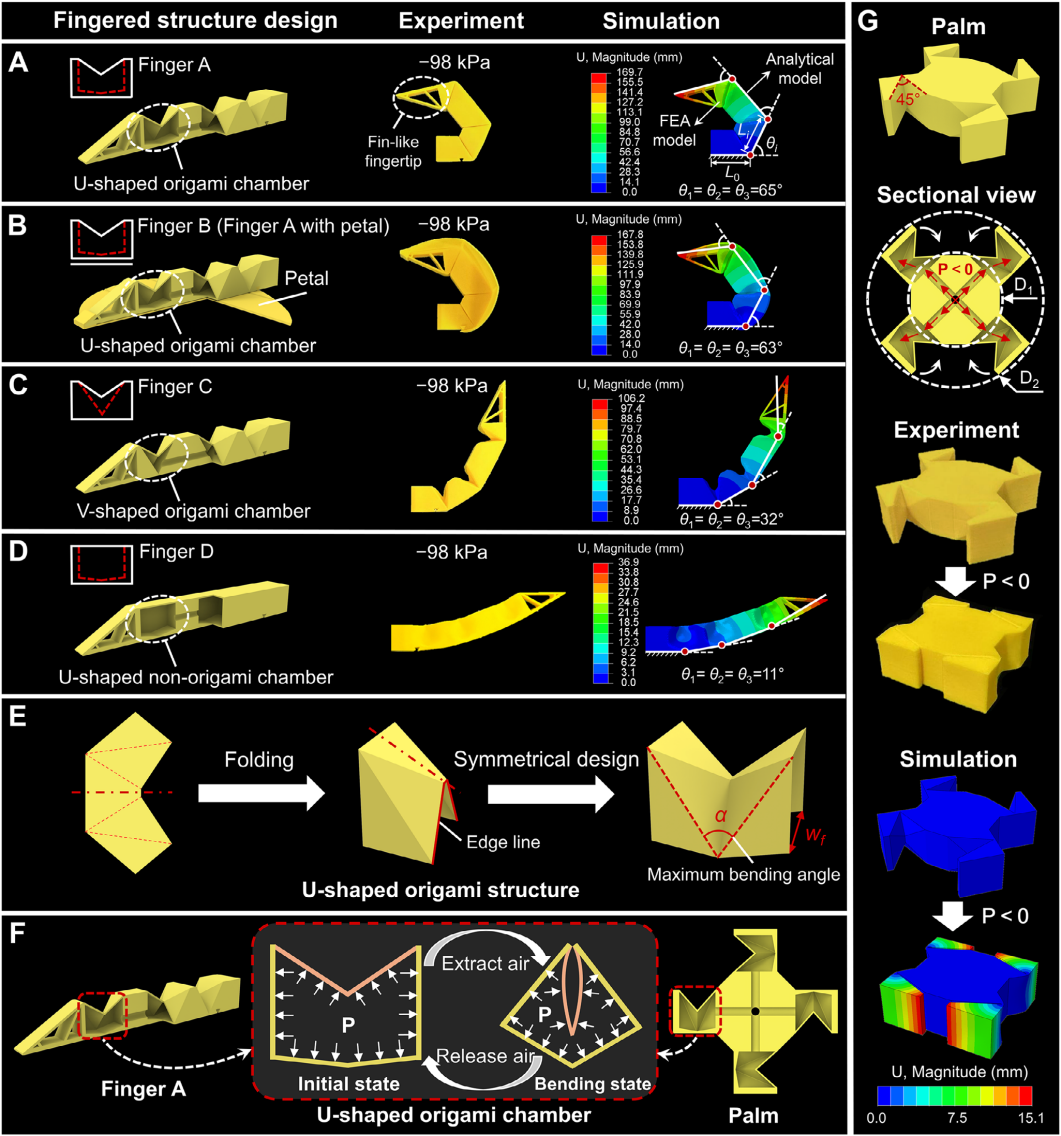
Fingers and palm of the SRSG. (A−D) Experimental, simulation, and theoretical results for four fingers under −98 kPa. Each finger consists of three chambers (functioning as joints) and four solid segments (analogous to human phalanges and serving as linkages); the base segment is fixed to the central palm, whereas the remaining three are movable. (A) Finger A with a U‐shaped origami chamber. (B) Finger B with a U‐shaped origami chamber integrated with a petal. (C) Finger C with a V‐shaped origami chamber. (D) Finger D with a U‐shaped chamber featuring flat rectangular surfaces. (E) Folding process of the U‐shaped origami chamber. The planar pattern is folded along the red dashed lines into a three‐dimensional structure with five surfaces. When the two outermost surfaces become parallel (separated by *w_f_* = 18 mm) and perpendicular to the ground, half of the chamber is formed. Following the symmetry principle, mirroring along the rightmost edge lines to obtain the complete origami chamber. (F) Schematic illustration of the bending deformation for the U‐shaped origami chamber. (G) Structural design of the central palm and comparison between experimental and simulation results. U‐shaped origami chambers are also incorporated at the four joints of the palm.

The comparative analysis of simulation and experimental results shows that the petals exert minimal influence on finger bending, as evidenced by a relative displacement error of only 1.1% between Fingers A and B. This marginal effect arises from the three trapezoidal grooves on the petal surface, which help mitigate interference with bending deformation while preserving structural rigidity. In contrast, the chamber architecture has a much more substantial impact on deformation behavior. The tip displacement of Finger A exceeds that of Finger C by 46.2%, with nearly twice the bending angle. This difference stems from the 67.3% smaller internal chamber volume of the V‐shaped chamber in Finger C compared with the U‐shaped chamber in Finger A, which prevents full contact between adjacent chamber surfaces even at −98 kPa. Similarly, the maximum tip displacement of Finger A is 4.6 times that of Finger D, with the joint bending angle approximately 5.9 times greater. This enhancement arises because a creased chamber joint, unlike a crease‐free one, bends preferentially along the crease lines, reducing structural stiffness, thereby enhancing bending performance. Thus, increasing the effective chamber volume within a suitable range can enhance the bending deformation capability of the finger, while the surface creases guide the chamber joint to fold along the preset crease lines and reduce bending deformation resistance, providing a basis for subsequent chamber joint modeling. Based on the above structural comparisons, the U‐shaped origami chamber was selected as the joint structure of the SRSG.

To further verify the origami chamber structure design, we established an analytical model for the representative U‐shaped origami chamber joint. In this model, the theoretical relationship between the joint rotation angle and the internal chamber volume variation is derived through geometric analysis. Furthermore, the pressure versus torque of the chamber joint is obtained based on the principle of work equilibrium (Text S4 and Figure S8). This model provides a detailed analysis of how structural parameters influence the folding‐induced rotational deformation of the origami chamber joint. Figure 2E illustrates how a centrally symmetric planar structure transforms into a three‐dimensional U‐shaped origami structure through sequential folding along predefined crease lines. Upon applying negative pressure, an attractive force develops within the origami chamber, drawing the opposite sidewalls toward each other, and thereby inducing bending deformation at the chamber joint until a stable contact state is established. Once the negative pressure is released, the chamber rapidly returns to its original configuration owing to the intrinsic elastic recovery of the TPE material (Figure 2F). Finite element simulations show that when the applied negative pressure exceeds approximately 70 kPa, the opposite sidewalls of the U‐shaped chamber come into contact, forming a stable contact state (Figure S9). Beyond this threshold, further increases in negative pressure yield little further bending, causing the bending angle to plateau at a saturated value α. This explains why the bending angles of Finger A and Finger B differ markedly below this pressure but converge progressively above it. Leveraging this characteristic, we customized U‐shaped origami chambers at the four joints of the central palm, with the maximum bending angle tailored to 45° (Figure 2G). This design ensures a robust transition between parallel and diagonal configurations of the SRSG by actuating four chamber joints of the palm, enabling reversible self‐reconfiguration of the gripper.

Furthermore, to address the challenge that existing soft grippers struggle to reconcile precise pinching with compliant grasping [41], we developed a fin‐like fingertip design (Figure S10). Unlike a conventional solid fingertip, this design enables an adaptive transition between two contact modes (i.e., pinching contact and conforming contact), effectively alleviating stress concentration typically observed in solid fingertips while retaining sufficient structural strength. Finite element simulations were conducted to compare the contact responses of two fingertip designs (i.e., the fin‐like and solid fingertips) when interacting with objects at two different positions (Figure S11). These results inform the structural optimization of the fingertip, allowing the SRSG to achieve adaptive grasping through physical intelligence without relying on local sensing or sophisticated control strategies.

### 2.3 Performance Characterization of the Fingers and Palm

The mechanical performance of the fingers was evaluated in terms of response time, bending deformation, tip trajectory, and output force (Figure 3). As shown in Figure 3A, the response time follows a clear hierarchy: the palm responds most rapidly (∼130 ms), followed sequentially by Finger A (∼211 ms), Finger B (∼309 ms), Finger C (∼445 ms), and Finger D (∼605 ms). The discrepancy primarily arises from three factors: (i) an increased number of internal chambers lengthens the airflow path and delays pressure transmission; (ii) variations in internal chamber volume and crease geometry affect airflow velocity; and (iii) the inherent mass and stiffness of the petals, coupled with passive bending behavior, collectively reduce actuation efficiency. Addressing these factors provides a viable pathway for further enhancing response speed. To further characterize the temporal response of the SRSG, we measured the complete temporal profiles of the representative Finger A and the central palm during one bending and recovery cycle (Figure S12). Considering transient oscillations in the actuation response, the complete cycle time is determined based on the stable bending state and the completely recovered state. Accordingly, one complete bending and recovery cycle is approximately 0.541 s for Finger A and 0.38 s for the central palm, corresponding to operational frequencies of about 1.85 and 2.63 Hz, respectively.

**FIGURE 3:**
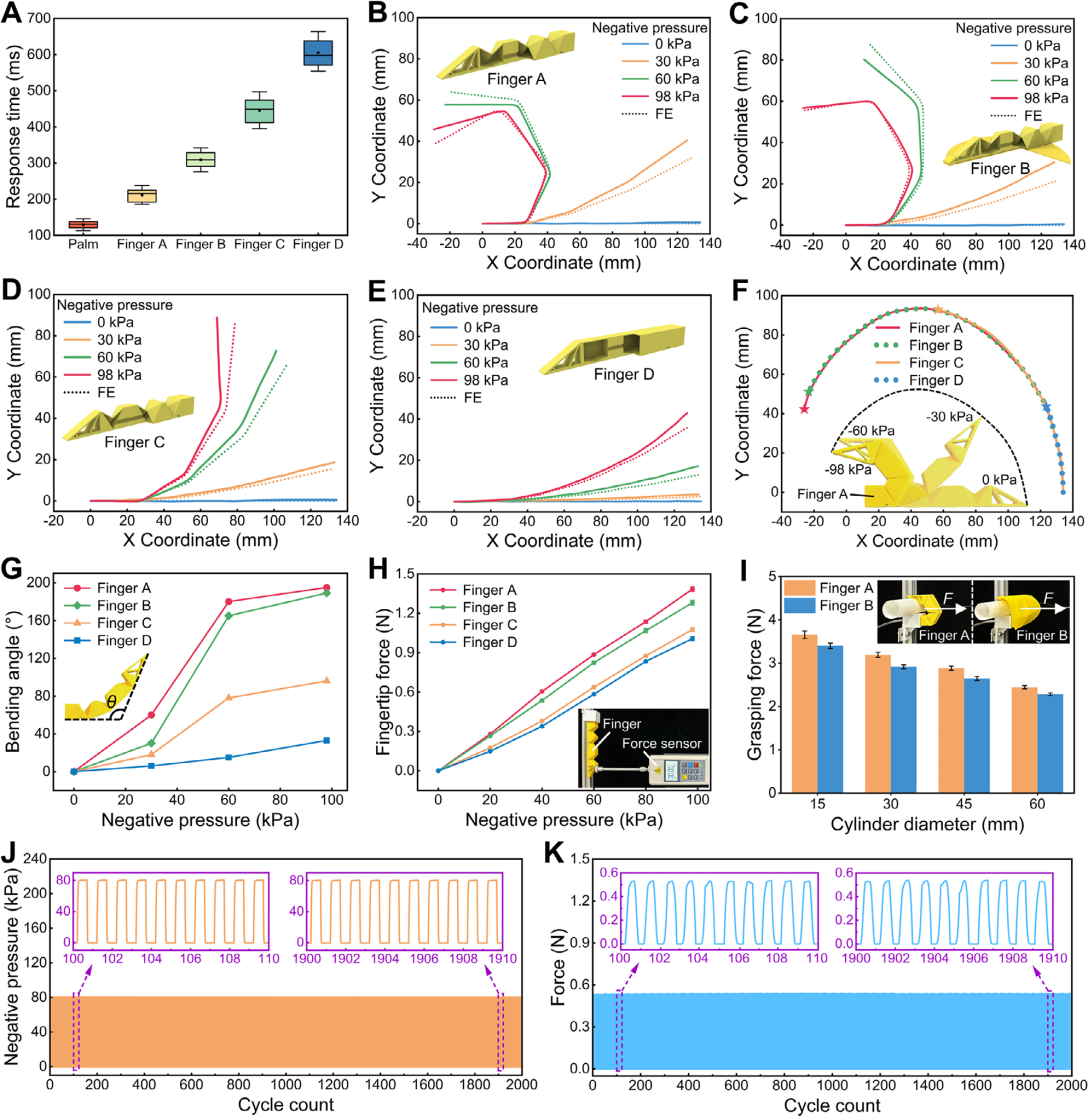
Characterization of response time, bending deformation, tip trajectory, and output force of four fingers. (A) Response time to maximum bending deformation for the central palm and four fingers. (B−E) Finite element simulations and experimental results of finger bending deformation under different negative pressures (0, −30, −60, and −98 kPa). (F) Tip trajectories of the four fingers. (G) Bending angle versus applied negative pressure. (H) Fingertip force versus applied negative pressure. (I) Comparison of grasping forces for Fingers A and B when embracing cylinders with diameters of 15–60 mm. The orange bars denote Finger A (without petals), and the blue bars denote Finger B (with petals). The results for (A, F, I) are obtained at −98 kPa. (J) Negative pressure and (K) Output force of Finger A during 2000 vacuumizing and releasing cycles at −80 kPa.

To comprehensively assess the bending performance and grasping range of the fingers, we conducted finite element simulations and experiments under four different negative pressure levels (Figure 3B−E), and the results exhibit consistent trends. In addition, based on the established kinematic model, we calculated the tip trajectories of the four fingers and compared them with experimental data, revealing excellent agreement under unloaded conditions (Figure 3F and Figure S13). As the external tip payload increased, the experimentally measured tip trajectories progressively deviated downward, mainly along the *y* direction, while the variation along the *x* direction was relatively limited. Taking Finger A as a representative example, in the initial unactuated state, the maximum downward offset along the *y* direction was about 4.2 mm under a 10 g load and increased to approximately 24.3 mm under a 50 g load. During the bending process of the finger, the maximum trajectory deviation along the *y* direction was roughly 3.4 and 15.4 mm under 10 and 50 g loads, respectively. This deviation can be explained by a cantilever‐beam‐like loading effect, i.e., the tip payload induces a downward displacement of the soft finger. The nonuniform stretching deformation of the TPE material leads to uneven joint rotations, causing the loaded finger to no longer strictly satisfy the equal joint angle assumption used in the analytical model. However, after being fully driven by a negative pressure of 98 kPa, the final fingertip positions under the three loading conditions of 0, 10, and 50 g remained nearly identical. We also measured the bending angle of all fingers under varying negative pressure (Figure 3G). Collectively, these results indicate that Finger A achieves the largest bending amplitude and thus the widest grasping range, whereas Finger D exhibits the weakest performance.

Subsequently, we quantified the output forces of four fingers by pressing their tips vertically against a force gauge under different negative pressures (Figure 3H). The fingertip force increases approximately linearly with the applied negative pressure, with Finger A generating the largest force. Given the limited bending performance and grasping range of Fingers C and D, which struggle to embrace the cylindrical objects, only Fingers A and B were selected for grasping force tests (Figure 3I). The experimental setup is shown in Figure S14, and detailed procedures are provided in the Experimental Section. We measured the grasping force for four cylinders with diameters ranging from 15 to 60 mm in 15 mm intervals. The results show that the grasping force decreases with increasing cylinder diameter, as a larger diameter increases the finger bending radius, reducing the contact area between the finger and the cylinder, and thus diminishing the grasping force. Moreover, Finger B exhibits a slightly lower grasping force than Finger A, which is attributable to the lack of active bending in the petals, leading to additional resistance during finger deformation.

Taken together, these results identify Finger A as the most suitable finger design for the SRSG, while the petal modules serve as a functional complement in specific grasping scenarios. Although the petal introduces a modest performance trade‐off, this cost is far outweighed by its enveloping grasping function. To further evaluate the durability of the selected finger in practical operation, cyclic durability tests were conducted on Finger A, as shown in Figure S15. The detailed test procedure is provided in the Experimental Section. During the test, the FDM 3D‐printed thin‐walled origami chamber exhibited high compressive robustness under repeated vacuum actuation. At −80 kPa, Finger A maintained stable output force and good airtightness during 2000 vacuumizing and releasing cycles, with no obvious performance degradation observed (Figure 3J,K). These results demonstrate that the thin‐walled chamber has excellent repeatability and durability, thereby satisfying the basic requirements for practical applications.

### 2.4 Mechanical Characteristics of the SRSG

By combining diagonal and parallel finger arrangements with detachable petals, the SRSG can be formed into four distinct configurations, namely Grippers I−IV (Figure 4A–D). Each configuration provides structural adaptation for different object geometries and manipulation requirements. Gripper I is preferred for circular, spherical, and irregular items because the diagonal finger arrangement has better geometric closure and more uniform contact force distribution. Gripper II is suitable for enveloping multiple scattered targets, as the petals create a nearly enclosed space that prevents them from falling out during transport. Gripper III is suited for elongated and plate‐like objects, since the parallel finger arrangement offers a larger effective contact area and higher horizontal resistive force. Gripper IV is regarded as a functional extension of the parallel grasping configuration; when the petals are attached, the staggered overlapping petals can improve the grasping stability. In addition, the petals effectively broaden the fingers, bridge the gaps between adjacent fingers, and increase the contact area with targets, which is beneficial for handling elongated objects and reducing slipping risk during transferring. Finite element simulations faithfully reproduce the closing and self‐reconfiguration behavior of Grippers I−IV, showing good agreement with experimental results and providing a reliable basis for design optimization of soft grippers. To assess the effects of finger arrangements and petals on grasping performance, we systematically characterized the mechanical properties of Grippers I−IV. Using an electronic force sensor, we measured the maximum grasping forces of the four grippers when grasping spheres, cylinders, and flat plates of various sizes (Figure 4F–I and Figure S16).

**FIGURE 4:**
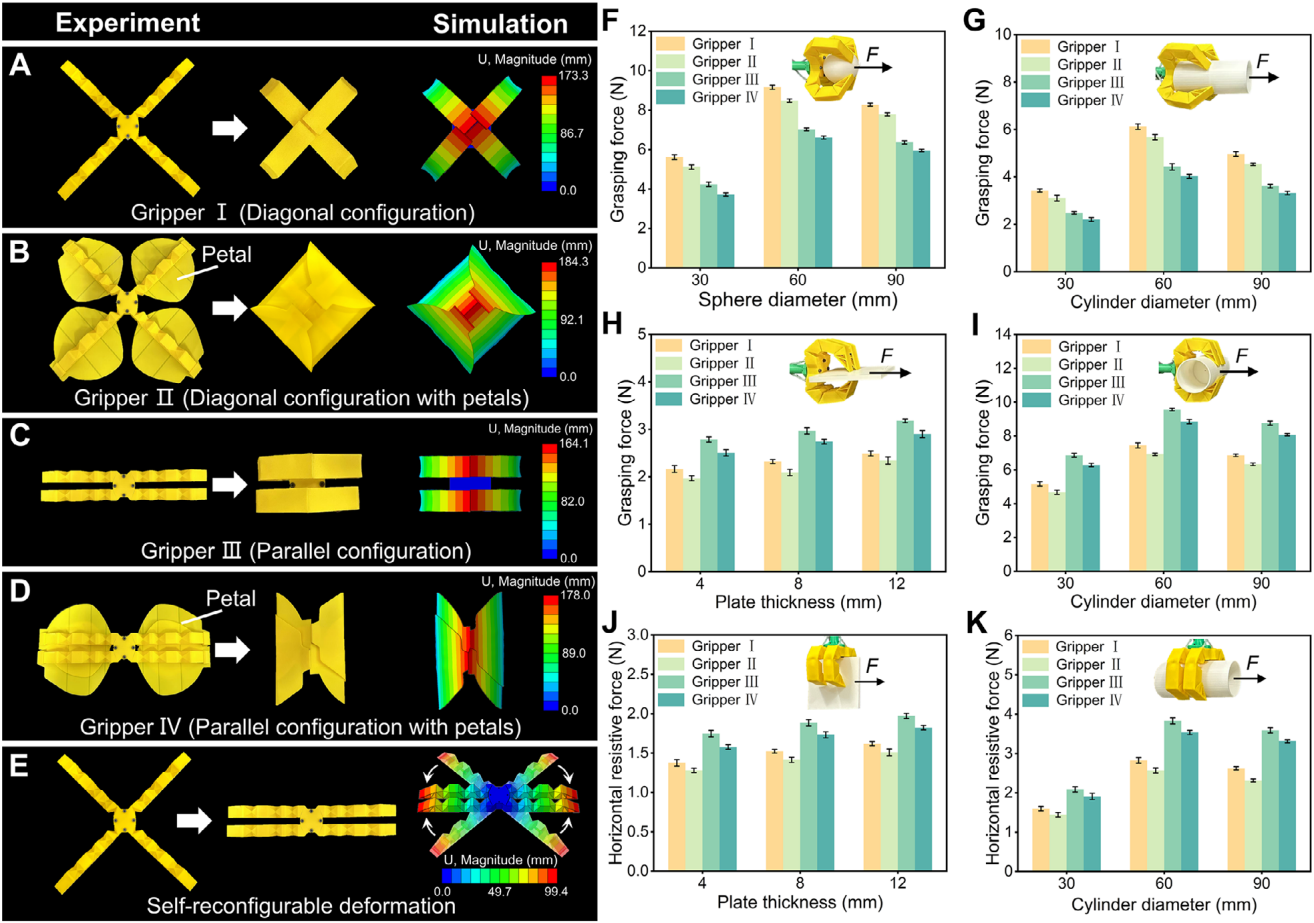
Performance characterization of the SRSG in different configurations. (A−D) Experimental and simulation results for the closing process of four soft gripper configurations (top view). (A) Gripper I (diagonal finger arrangement). (B) Gripper II (diagonal finger arrangement with petals). (C) Gripper III (parallel finger arrangement). (D) Gripper IV (parallel finger arrangement with petals). (E) Self‐reconfigurable deformation of the SRSG. (F−I) Maximum grasping forces of the SRSG when grasping spheres and cylinders of varying diameters, and plates of varying thicknesses. (J, K) The maximum horizontal resistive force of the SRSG when grasping plates of varying thicknesses and cylinders of different diameters. All results are obtained at −98 kPa.

When grasping spheres and cylinders parallel to the gripper's axis (Figure 4F,G), Gripper I exhibits the highest grasping force, averaging 34.4% greater than that of Gripper III. This enhancement arises from the diagonal finger arrangement, which allows fingers to have a larger contact area with the object and promotes a more uniform distribution of contact points. By contrast, when grasping flat plates and cylinders oriented perpendicular to the gripper's axis (Figure 4H,I), Gripper III outperforms Gripper I by an average of 28.9%, as the parallel arrangement offers a larger effective contact area with rectangular objects than the diagonal arrangement. We further quantified the horizontal resistive force of four grippers during grasping. Gripper III exhibits 31.9% higher horizontal resistive force than Gripper I when grasping plates and cylinders (Figure 4J,K), which aligns with the principle that the parallel arrangement enables the fingers to form a larger contact area with rectangular cross‐section objects, thereby increasing frictional force. Grippers II and IV (with petals) exhibit the same performance trends, and detailed comparisons are omitted here for brevity.

We further compared the output forces of the two‐finger and four‐finger grippers during object grasping (Figure S17). The results show that the four‐finger gripper produces approximately twice the output force of the two‐finger counterpart, primarily owing to its larger effective contact area and more support points. Consequently, for objects with different cross‐sectional geometries, employing the four‐finger gripper with an appropriate finger arrangement not only substantially enhances grasping force and horizontal resistive force but also increases contact area with the object, thereby improving grasping stability and success rate.

It is worth noting that both the grasping force and horizontal resistive force are affected by object size (Figure 4F–K and Figure S17). When the object size falls within the range of the palm's inner and outer diameter (D1−D2, 50−70 mm, Figure 2G), the gripper achieves maximal surface contact with the object, thereby maximizing the effective contact area and producing peak output forces. Outside this range, the output force decreases markedly due to reduced contact area, leading to diminished grasping performance.

### 2.5 Adaptive Bidirectional Rotation of Fragile Items

Previous studies have reported two primary strategies for enabling soft grippers to rotate objects. The first relies on finger bending to secure the object, followed by lateral deformation to induce rotation [42, 43]; however, this method suffers from low efficiency, limited torque output, frequent slippage, and complex control. The second employs self‐twisting origami structures to generate rotational motion [44, 45], which, while straightforward, only permits discontinuous rotation, severely restricting continuity of operation, thereby significantly reducing operational efficiency. In stark contrast to the above two methods, the SRSG achieves robust and continuous bidirectional rotation through self‐reconfigurable deformation. The process of rotating an object using the SRSG involves several sequential steps (Figure 5A). First, the gripper is positioned above the object at an appropriate location. Negative pressure is then applied to one pair of fingers (A1−A2 or B1−B2), inducing bending deformation to hold and secure the object. Subsequently, applying negative pressure to the central palm actuates the four chamber joints and generates rotational deformation to reconfigure the finger arrangements, thereby enabling clockwise or counterclockwise rotation of the object. Once the object reaches a stable orientation, the fingers release holding states. Following deflation, both the palm and all fingers return to their initial configurations, accomplishing a full rotation cycle. The control system architecture of the SRSG is shown in Figure S18, and the detailed control procedure is described in the Experimental Section. Unlike rotation operations achieved through simply driving the robotic arm's end joint, in our experiment, the actual rotation of the bulb is accomplished directly by the finger self‐reconfigurable deformation, whereas the robotic arm end only serves to fix and position the SRSG. Therefore, this continuous bidirectional rotation task demonstrates the SRSG's dexterous in‐hand manipulation capability, which is particularly useful for robotic arms with limited wrist rotation or without continuous end joint rotation. This capability enables the gripper to rotate objects independently, reducing dependence on the robotic arm's end joint rotation and offering an alternative strategy for rotation‐constrained manipulation scenarios. This actuation strategy is closest to the natural way that humans rotate objects with fingers, where holding and rotating actions are largely decoupled. Moreover, because the tip trajectory of the finger approximates a circular path (Figure 5B), once the gripper establishes stable holding, rotational motion is efficiently transmitted to the object, substantially mitigating slippage and enhancing rotational stability.

**FIGURE 5:**
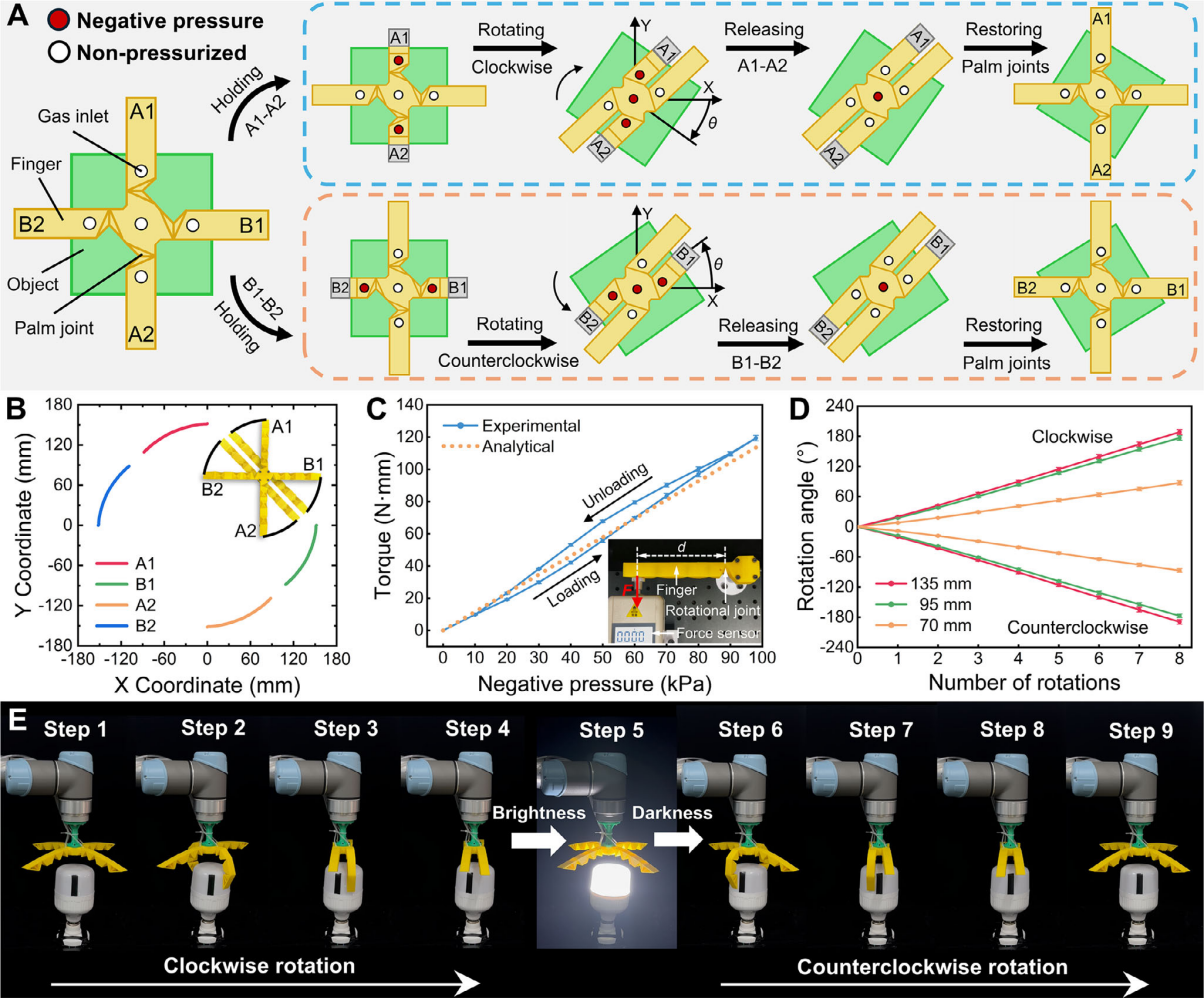
Adaptive bidirectional rotation of bulbs by the SRSG. (A) Schematic of the object rotation mechanism. Clockwise rotation by fingers A1−A2; counterclockwise rotation by fingers B1−B2. (B) Tip trajectories of the four fingers during self‐reconfiguration. (C) Output torque versus negative pressure. (D) Rotation angle versus number of rotations for the bulbs of different diameters under clockwise and counterclockwise rotation. (E) Continuous rotation sequence of a bulb. The clockwise rotation sequence includes: (i) mounting the SRSG at the end of the UR5 robotic arm and positioning it above the bulb; (ii) applying negative pressure to actuate fingers A1−A2 for stable holding the bulb; (iii) actuating the palm to reconfigure fingers A1−A2, thereby rotating the bulb clockwise; (iv) releasing fingers A1−A2 after finishing one rotation step; and (v) returning the palm and all fingers to their initial configuration, accomplishing one complete clockwise rotation cycle. The counterclockwise rotation process follows the same procedure, with fingers A1−A2 replaced by fingers B1−B2.

To characterize the rotational force of the fingers, we measured the output torque of a single finger, which can be theoretically expressed as *T* = *Fd*, where *F* is the blocking force generated when the palm's rotational joint drives the finger to press against the force sensor, and *d* is the distance from the force measurement point to the rotation center of the joint. In addition, based on the output torque model of the origami chamber joint (Text S4), we calculated the theoretical relationship between torque and pressure and compared it with the experimental result, revealing the same rising trend as the negative pressure increases (Figure 5C). The experimental loading and unloading curves generally agree with the analytical ones. During the loading phase, the torque increases approximately linearly with the negative pressure. During unloading, the experimental torque is slightly higher than the theoretical one due to the hysteresis effect of pressure release. At −98 kPa, the measured peak torque reaches approximately 121 N·mm, while the theoretical value is 114 N·mm, yielding a relative error of 6.1%. This discrepancy primarily stems from simplifications in the theoretical model and the pressure hysteresis. To further evaluate the SRSG's performance, we compared it with representative rotary actuators (Table S3) [42, 43, 45−47]. The comparison results show that the SRSG has higher torque, faster response, and continuous bidirectional rotation, demonstrating the practical significance and advantage of its dexterous in‐hand rotation manipulation.

By integrating self‐reconfigurable deformation with the fin‐like fingertip design, the SRSG enables continuous, bidirectional rotation of fragile objects such as bulbs with diameters ranging from 70 to 135 mm (Figure S19). It can be found that the larger the diameter of the bulb, the greater the rotation angle, with an average maximum rotation angle of approximately 24° per actuation, and clockwise and counterclockwise rotation angles are nearly exactly symmetrical (Figure 5D). This size‐dependent behavior arises from the fact that bulbs with smaller diameters possess higher curvature, which increases the gap between the bulb and the fingers (Figure S20A), thereby reducing the effective contact area. Moreover, the curved bulb surface provides only limited contact with the fingertip, making it more susceptible to slippage. For a given amount of slippage, it generates a relatively larger gap between a smaller diameter bulb and the finger (Figure S20B), further reducing the contact area and available frictional force. As a result, for a larger bulb, it enables a greater rotation angle and higher rotational stability. Through multiple consecutive and reversible rotation cycles, the SRSG rotates the bulb clockwise from its dark, switched‐off state to a bright, switched‐on state, and then counterclockwise back to the initial orientation (Figure 5E and Movie S5). Repeating this cycle enables periodic, stable, and controllable rotation motion of the bulb. This mechanism ingeniously converts the self‐reconfiguration movement of the fingers into efficient object rotation. It not only enhances the gripper's dexterity but also introduces a novel strategy for precise and repeatable rotation manipulation.

### 2.6 Enveloping Capture of Multiple Fine Particles and Live Aquatic Organisms

Inspired by the overlapping closure mechanism of rapeseed flowers, we designed Gripper II with petals that transform the initially open grasping region into an almost sealed space upon closure, thereby enabling efficient and stable capture of multiple objects in both atmospheric and underwater environments. To clarify the contribution of the petals to enveloping grasping performance, we conducted comparative experiments using Gripper I (without petals) and Gripper II (with petals) to wrap and grasp multiple fine particles (rice grains with a diameter of 1 mm and soybeans with a diameter of 4 mm) under an identical insertion depth (*h* = 80 mm) (Figure 6A–D and Movie S6). The results clearly show that Gripper II not only markedly outperforms Gripper I in terms of the number of grasped rice grains and soybeans each time but also reduces particle loss during lifting and transferring, indicating superior enveloping capability of the petals. This performance advantage stems from the much larger enclosed volume of Gripper II, which is approximately eight times that of Gripper I (detailed calculations in Text S5 and Figure S21), substantially improving its ability to grasp fine and small‐scale particles in bulk.

**FIGURE 6:**
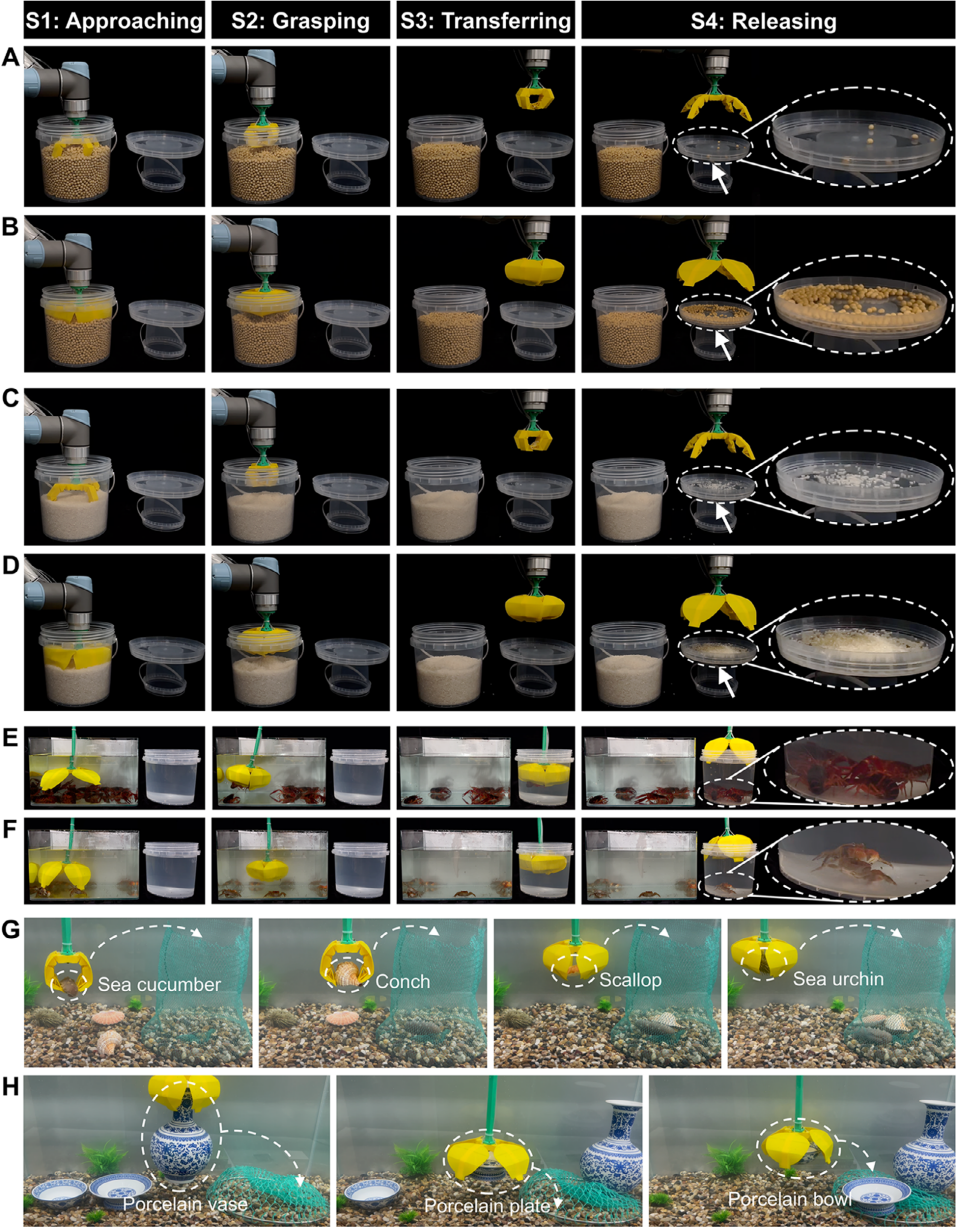
Enveloping grasping performance of the SRSG in air and underwater. (A−D) Comparison of Gripper I (without petals) and Gripper II (with petals) during the grasping of soybeans and rice grains. The grasping sequence includes: (i) inserting the gripper into the bucket to a prescribed depth using a UR5 robotic arm; (ii) closing the petals to envelop the target particles; (iii) transferring the enclosed particles above a circular tray; and (iv) releasing the particles to complete the grasping task. (E, F) Underwater capture of multiple swimming crayfish and crabs in a single operation. The procedure involves approaching the organisms, closing petals to secure them, lifting them to the designated position, and releasing them into a plastic bucket. (G) Continuous underwater capture of different creatures, including a sea cucumber, conch, scallop, and sea urchin. (H) Continuous underwater handling of diverse porcelain items, involving a vase, plate, and bowl.

We further evaluated the gripper's ability to capture live aquatic organisms prone to stress responses. Gripper II exhibits several functional advantages in this context: a large enclosed volume to accommodate multiple organisms simultaneously, excellent sealing performance to prevent escape, an ultrashort closing response time that enhances grasping success rate, and inherent hydrophobicity that mitigates water leakage during prolonged submersion. As a result, Gripper II can reliably capture multiple stress‐responsive crayfish and crabs in a single underwater operation (Figure 6E,F, and Movie S7), which stands in sharp contrast to conventional soft grippers that typically capture only one organism at a time [31, 32, 48]. This improves operational efficiency to a great extent and presents a promising approach to efficient underwater harvesting. Since Gripper II is entirely fabricated from soft TPE 85A, it enables gentle, non‐destructive capture and release of crayfish and crabs, which resume normal swimming behavior immediately after being released. As shown in Figure 6G,H, and Movie S8, the petals further expand the practical utility of the SRSG for continuous underwater capture of diverse objects, including underwater creatures such as soft sea cucumbers, smooth‐shelled conchs and scallops, spiny sea urchins, and fragile porcelain items such as a vase, plate, and bowl. For these targets, the petal‐assisted enveloping design helps bridge the gaps between adjacent fingers and provides protective buffering during capturing and transferring, rather than relying solely on localized clamping or pinching, thereby reducing local stress concentration, slipping risk, and collision‐induced damage. These results demonstrate that the SRSG with petals enables stable and non‐destructive underwater capture of fragile, soft, irregular, sharp, and difficult‐to‐confine targets, highlighting its potential and practical utility for non‐invasive sampling of live organisms and fragile objects in complex, turbulent environments such as rivers and shallow water.

### 2.7 Multimodal Grasping of Fruits and Vegetables

Figure 7 demonstrates the multimodal grasping capabilities of the SRSG. Benefiting from the fin‐like fingertip design, the gripper provides sufficient local compliance at the contact surface, enabling adaptive grasping of fruits with diverse sizes, shapes, and surface textures without visible surface damage (Movie S9). Although the overall compliance of this 3D‐printed SRSG is inferior to that of traditional silicone‐based grippers, the flexible fingertip design mitigates this limitation to some extent. In the parallel grasping mode, Gripper III can precisely pinch a loquat stem with the fingertips’ tip and achieve conformal grasping of the banana and extremely soft durian pulp via the fingertips’ midsection (Figure 7A). In the diagonal grasping mode, Gripper I pinches small, hard‐shelled longans and lychees using the fingertips’ tips and conformally grasps mangoes with irregularly curved surfaces by the fingertips’ midsection (Figure 7B). In the enveloping grasping mode, Gripper II can securely lift clusters of small fruits (e.g., tangerines, cherry tomatoes, and grapes) without any fruit loss during transferring (Figure 7C). This robust enveloping capability is unattainable for Gripper I or III, emphasizing the value of reconfigurable finger arrangements and petal integration for handling multiple soft fruits in practical settings.

**FIGURE 7:**
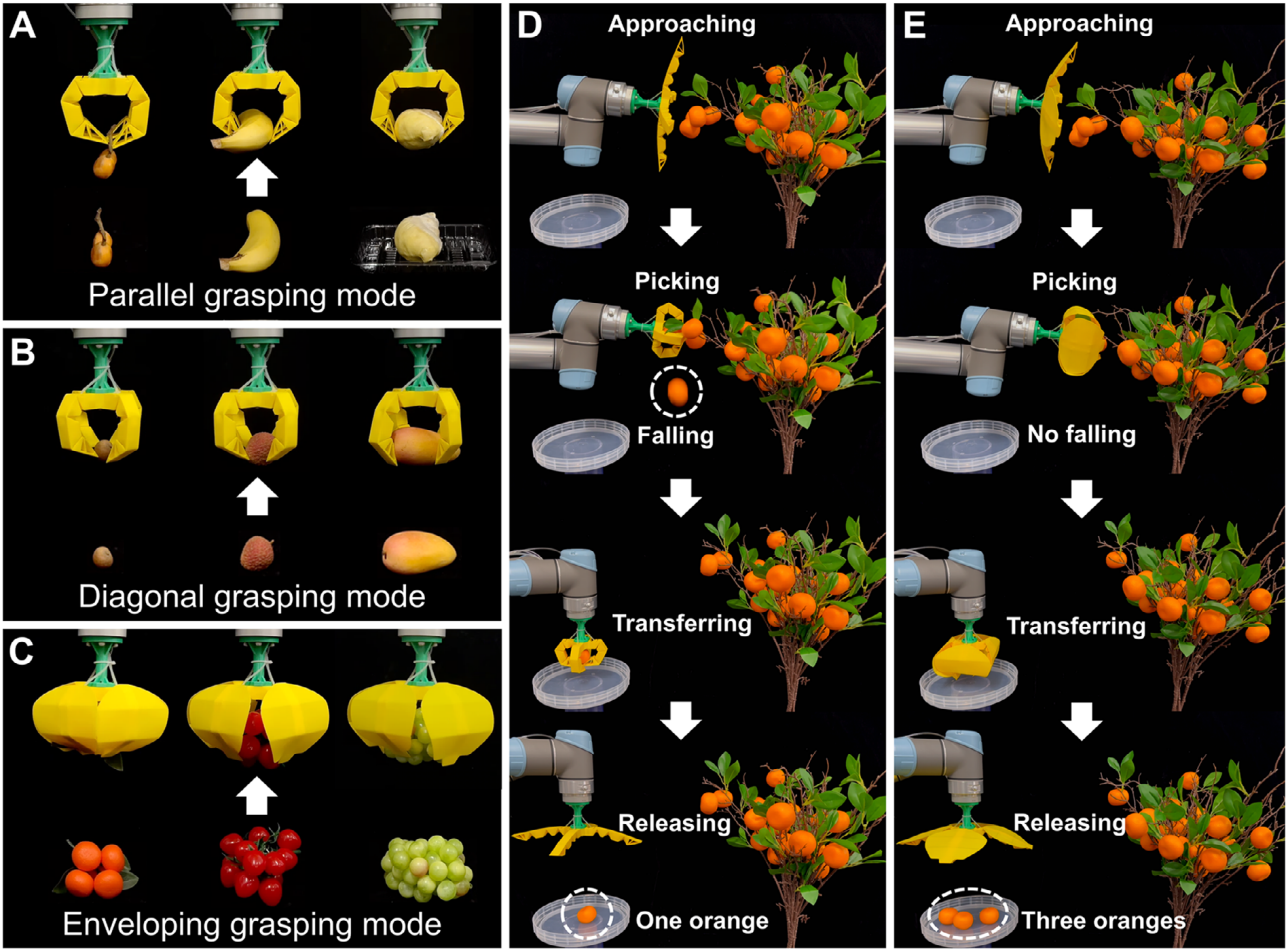
Multimodal grasping of various fruits using the SRSG. (A) Parallel grasping mode: precisely picking a loquat stem and conformal grasping of a soft banana and durian pulp. (B) Diagonal grasping mode: accurately manipulating a longan and a lychee, and adaptive grasping of an irregularly shaped mango. (C) Enveloping grasping mode: securely lifting clustered small fruits, including tangerines, cherry tomatoes, and grapes, without fruit loss or damage. (D, E) Process of picking oranges using Gripper I (without petals) and Gripper II (with petals), respectively.

We performed orange picking experiments using Gripper I and Gripper II to demonstrate the role of the petals in enveloping performance during fruit picking (Figure 7D,E, and Movie S10). The results show that Gripper II successfully envelops and picks three oranges in a single operation, and then transfers and places them onto a tray. By contrast, although Gripper I initially picks up two oranges, it can only retain one, with the other slipping out during transferring, resulting in only one orange being placed on the tray. This clearly demonstrates that the SRSG equipped with petals exhibits excellent enveloping and protective performance during fruit picking and transferring.

To evaluate the continuous operational performance of the SRSG on soft objects, we selected six representative fruits and vegetables (longan, lychee, apple, grape, chili, and bananas), placed them at predefined locations, and then used the SRSG to sequentially grasp and relocate them onto a tray (Figure S22 and Movie S11). Throughout the continuous manipulation, the SRSG successfully handled all items without causing damage, owing to its compliant fin‐like fingertips and self‐reconfigurable design, which enable dynamic adaptation to the targets’ geometry. This multifunctional, bio‐inspired gripper flexibly switches among three grasping modes and enables continuous manipulation of objects spanning a wide range of sizes, shapes, quantities, masses, and hardness levels. With these capabilities, the SRSG emerges as a promising candidate to progressively supersede traditional soft grippers restricted to a single grasping mode, thereby broadening the research frontiers of soft robotic grippers [18, 49]. Detailed dimensions and weights of all tested fruits and vegetables are summarized in Table S4.

### 2.8 Large‐Range Continuous Cross‐Scale, High‐Load Grasping Performance

The cross‐scale grasping performance is crucial for enhancing the flexibility, versatility, and adaptability of soft grippers. In this work, the object size reported in cross‐scale grasping tasks refers to the effective grasping size, i.e., the actual dimension of the object directly contacted and constrained by the gripper during manipulation, rather than its maximum overall dimension. Such capability can reduce or even eliminate the need for task‐specific fixtures or frequent manual gripper replacement, thereby improving operational efficiency, cutting costs, and broadening application scenarios. In conventional finger‐type soft grippers, the fingers typically remain perpendicular to the palm in the unactuated state [12, 18, 19, 20], which fundamentally limits the maximum attainable grasping range. By contrast, in the proposed SRSG, the four fingers and palm lie in the same plane in the initial state, and this layout not only essentially enlarges the accessible grasping range but also supports robust continuous operation and high load capacity, outperforming most conventional soft grippers that suffer from narrower operational ranges and lower load capacities (Figure 1C and in Table S5) [12, 13, 14, 15, 16, 18, 19, 20, 22, 31, 32, 39, 49, 50, 51, 52, 53, 54, 55, 56]. Moreover, the self‐reconfiguration mechanism, detachable petal modules, and fin‐like fingertip design of the SRSG collectively broaden its cross‐scale grasping range across objects of different sizes by changing the finger arrangement, extending the grasping workspace, and adaptively switching between pinching contact and conforming contact.

We evaluated the SRSG's grasping success rate and stability by conducting grasping tests on eight representative objects. The results show that the gripper can actively adjust its configuration to conform to the contours of each object, thereby improving the success rate (Table S6). Grasping stability tests further show that the SRSG can securely hold a large object (a plastic bucket containing a football) while undergoing 180° clockwise and counterclockwise rotations, performing a translational motion along a rectangular trajectory, and rotating around the end‐joint axis of a UR5 robotic arm. Through all three sets of tests, no objects were dropped, and only minimal vibration was observed, confirming excellent grasping stability (Figure S23 and Movie S12).

To assess the maximum manipulable range and the maximum payload capacity of the SRSG, we conducted dedicated tests for each metric independently (Movie S13). Leveraging the fin‐like fingertip, the SRSG can precisely pinch a single human hair as fine as 0.07 mm in diameter and securely grasp a square plate up to 270 × 270 mm in size (Figure 8A,B). Based on the effective grasping size, the smallest and largest manipulated objects span three orders of magnitude in size, with a scale ratio of approximately 3857. The SRSG successfully lifted a bracket loaded with weights of 5.6 kg (Figure 8C), equivalent to approximately 106 times its own weight (53 g), highlighting its exceptional load‐bearing capability.

**FIGURE 8:**
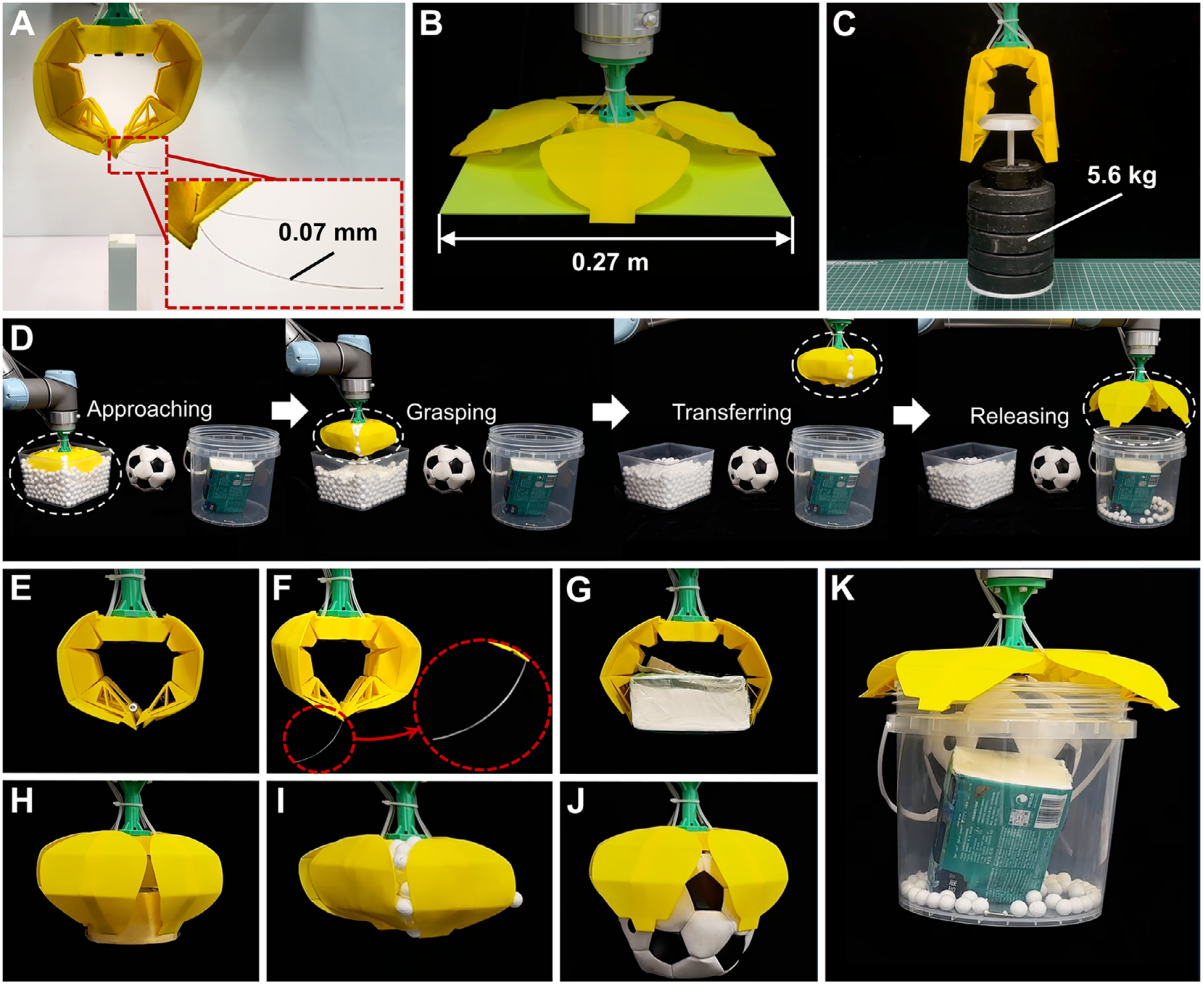
Cross‐scale, high‐load, multi‐particle integrated performance of the SRSG. (A) Pinching a human hair with a diameter of about 0.07 mm. (B) Grasping a square plate with dimensions 270 × 270 mm. (C) Lifting a bracket loaded with weights totaling 5.6 kg. (D) Enveloping and transferring a large quantity of foam balls into a plastic bucket. (E−K) Representative objects grasped by the SRSG: an M3 screw, a fluorocarbon line, a pack of tissues, a roll of single‐sided tape, multiple foam balls, a football, and a plastic bucket filled with all the above items.

We further evaluated the practical grasping performance of the SRSG by conducting continuous grasping tests on common items with varying shapes, sizes, quantities, and weights. Continuous grasping was implemented by pre‐programming a sequence of waypoints on the UR5 robotic arm's end effector. The initial positions of the items and the grasping sequence are shown in Figure S24 and Movie S14. Figure 8D illustrates the grasping process for a representative object. The SRSG can manipulate objects ranging from a lightweight M3 screw (Figure 8E) and a 0.2 mm diameter fluorocarbon line weighing less than 1 g (Figure 8F), to medium‐sized items such as a pack of tissues with a width of 130 mm (Figure 8G) and a 100 mm diameter single‐sided tape (Figure 8H), and further to large‐sized, heavy objects including a 170 mm diameter football (Figure 8J) and a 230 mm diameter plastic bucket (Figure 8K). The gripper also handles multiple granular items such as foam balls (Figure 8I). The SRSG thus overcomes a longstanding challenge that existing grippers struggle to integrate continuous operation, multi‐particle capture, cross‐scale manipulation, and high load capacity into a single monolithic soft system, enabling robust and multifunctional manipulation in complex unstructured environments.

## 3 Conclusions

Reconfigurability is a key functional characteristic of soft robotic grippers, driving the pursuit of the autonomous, efficient, and physically intelligent soft gripper system capable of executing advanced, complex grasping tasks. However, current reconfigurable soft grippers still rely on external rigid transmission mechanisms or manual intervention, lacking the ability to self‐reconfigure their finger arrangements. Moreover, most soft grippers are unable to actively modulate their workspace for batch or multi‐object grasping, which fundamentally constrains grasping efficiency and operational continuity while increasing structural complexity and manufacturing costs.

This work brings to light a self‐reconfigurable grasping paradigm. Inspired by the morphological reconfiguration and closure mechanism of the rapeseed flower, we propose a SRSG capable of reconfiguring its finger arrangements, enabling rapid and reversible switching between diagonal and parallel configurations, and altering its grasping workspace. The SRSG addresses a longstanding challenge in soft robotics: integrating cross‐scale, multi‐particle, and high‐load manipulation capabilities into a single monolithic soft gripper, that is, a capability that has remained out of reach for conventional designs. Following the concept of learning from nature, the SRSG establishes a new design strategy for soft grippers by merging multiple manipulation functions within a single architecture. This gripper autonomously self‐reconfigures into the appropriate configuration on demand, significantly enhancing both output force and grasping success rate while improving adaptability to objects with diverse contours. We further designed U‐shaped origami chambers that serve as the gripper's movable joints, achieving rapid reconfiguration (∼130 ms) and high joint output torque of approximately 121 N·mm. By combining self‐reconfigurability with a fin‐like fingertip design and detachable petals, the gripper enables efficient and continuous rotation of bulbs, stable enveloping of small particles such as rice grains and soybeans, non‐destructive underwater capture of multiple stress‐responsive live aquatic organisms and fragile porcelain items, and adaptive pinching and conforming of soft fruits and fine objects, including hairs and fluorocarbon line. These integrated capabilities enable dexterous, precise, and controllable manipulations, demonstrating remarkable advantages over existing soft grippers.

Compared with conventional tendon‐driven grippers, the origami chambers used in the present gripper are directly actuated by negative pressure, enabling a favorable combination of rapid grasping response, high output force, and simplified structural composition (Table S7) [12, 14, 57−61]. In particular, the proposed gripper exhibits a shorter response time than representative tendon‐driven grippers, largely because finger deformation is directly driven by vacuum‐induced folding of the origami chambers rather than tendon transmission, thereby avoiding the friction, compliance, and structural complexity commonly associated with tendon‐routing mechanisms. As a result, the SRSG achieves a rapid grasping response within 0.211 s, delivers a grasping force of approximately 56 N (about 106 times its own weight) with a self‐weight of only 53 g, and possesses low control complexity, thereby highlighting the superiority of the lightweight monolithic SRSG design. The entire device is monolithically 3D‐printed at a cost of less than US$1 per unit, challenging the conventional assumption that advanced robots are bound to be expensive. The SRSG exhibits a comprehensive grasping capability, enabling continuous manipulation of objects across a wide range of hardness, quantity, shape, and size. Its grasping diameter spans from 0.07 to 270 mm, achieving a remarkable cross‐scale ratio of up to 3857 times and covering three orders of magnitude in size. The gripper also delivers a maximum payload of 56 N (about 106 times its own weight). Leveraging the self‐reconfigurable grasping paradigm, these results underscore the superiority of the SRSG in terms of key performance metrics, including grasping force, response speed, joint output torque, and cross‐scale grasping range. As summarized in Table 1, the SRSG outperforms several representative soft grippers in overall multifunctionality, ranking among the most versatile soft grippers reported to date. Furthermore, the self‐reconfiguration design concept based on flexible origami joints could be extended to other classes of soft robots (e.g., soft crawling, flying, swimming, and jumping robots) [62, 63, 64, 65, 66, 67, 68], enabling them to better adapt to diverse and unstructured natural environments.

**TABLE 1:** Comparison of existing robotic grippers.

Gripper	Self‐reconfiguration	Cross‐scale grasping	Rotating objects	Bulk objects	Underwater application	Multiple moving animals
This work	**☆**	**☆**	**☆**	**☆**	**☆**	**☆**
Octopus‐inspired gripper [32]		√		√	√	
Suction‐type gripper [31]		√			√	√
Finger‐type gripper [43]			√			
Wrapping‐type gripper [16]				√		
Human‐like gripper [70]			√			
Variable stiffness gripper [42]			√			
Continuum‐type gripper [14]		√				
Foldable‐type gripper [45]			√			

Although the proposed SRSG exhibits substantial advantages, several limitations remain. First, compared with traditional silicone‐based grippers, the 3D‐printed SRSG has lower intrinsic compliance that may limit the handling of extremely soft objects, and the TPE material's relatively low friction coefficient can reduce the gripper's resistance to slippage. Second, the current system operates in open‐loop control and does not yet incorporate closed‐loop strategies, lacking the ability to autonomously adjust to target variations or regulate grasping force using real‐time visual‐tactile feedback such as machine vision or tactile sensing. Third, direct deployment of the present SRSG in deep‐sea environments remains challenging, mainly because the origami chambers contain compressible air. High ambient hydrostatic pressure would naturally crush the hollow origami chambers filled with air before vacuum actuation, causing the gripper to deviate from the predictable deformation behaviors observed in air or shallow water areas, which would seriously affect its deformation capability, structural stability, and operating controllability. Accordingly, the present gripper design is better suited to laboratory settings and shallow water environments rather than the deep‐sea region. Existing deep‐sea soft robotic systems typically cope with this challenge through pressure‐tolerant structural designs, the use of incompressible fluidic media such as seawater, or pressure‐compensated actuation strategies, which are inherently more appropriate for high hydrostatic pressure environments [10, 64, 69]. To address these challenges, future work will focus on developing high‐performance soft materials, integrating tactile sensors, variable stiffness structures, and closed‐loop control approaches for real‐time adjustment of grasping force and stiffness, and exploring pressure‐tolerant actuation strategies for operation under high hydrostatic pressure, thereby improving grasping stability and success rates. Additionally, embedding a depth camera into the gripper will allow autonomous identification of diverse objects, facilitating automatic selection of appropriate grasping modes to boost both adaptability and efficiency. Collectively, these research directions will advance soft grippers toward more diversified, multi‐scenario, and multi‐task applications, broadening the applicability of the SRSG across a wider range of unstructured environments.

## 4 Experimental Section

### 4.1 Fabrication and Assembly

Computer‐aided Design (CAD) models of the SRSG and petals were created in SolidWorks 2022 (Dassault Systèmes Inc.), with both the petals and U‐shaped origami chamber designed to have a uniform wall thickness of 0.8 mm. The models were then imported into Bambu Studio (v1.8.3, Bambu Lab) for slicing to generate the corresponding G‐code files, which were subsequently imported into a commercial FDM 3D printer (Bambu Lab P1S) for printing. The printing material is TPE 85A filament (Yasin3D), with a diameter of 1.75 mm. Before printing, the filament was dried in an oven preset at 80°C for 6 h. Detailed printing parameters are provided in Table S8. After fabrication, each petal was assembled by inserting its front hollow trapezoidal sleeve and rear dovetail joint into the corresponding fin‐like fingertip and dovetail groove of each finger.

### 4.2 Design of the Wall Thickness

The wall thickness of the finger chamber joints is a crucial design parameter for actual fabrication. Presently, while existing studies commonly use output force to evaluate the performance of 3D‐printed soft fingers [71, 72, 73, 74], we argue that the most meaningful metric should instead be the minimum printable wall thickness that maintains excellent airtightness. The reason is that force generation is fundamentally governed by internal pressure, and excessively thin walls are prone to air leakage, which directly reduces output force. Conversely, when airtightness is ensured, thinner walls increase structural compliance, enabling larger deformation and greater output force under the same applied pressure, ultimately improving grasping performance. Therefore, the minimum airtight wall thickness achievable via 3D printing serves as a key indicator of the soft fingers. In this work, the minimum printable airtight wall thickness is *T* = 0.8 mm.

### 4.3 FEA Simulation

The CAD models of the single finger and the complete SRSG were imported into commercial finite element software (Abaqus, Dassault Systèmes Inc.) to simulate bending deformation, and the results were validated against experimental measurements. A 10‐node quadratic tetrahedral hybrid element (C3D10H) was employed for meshing, improving solution accuracy without substantially increasing mesh density. Due to the near‐incompressibility of TPE 85A material, traditional finite element solutions tend to suffer from volumetric locking, resulting in the excessive stiffening effect. To address this issue, a hybrid formulation was adopted, effectively eliminating volumetric locking and enabling accurate prediction of large deformation behavior in soft materials. In the simulations, inelastic normal contact and frictionless tangential contact were defined on the inner and outer surfaces of the origami chamber, and negative pressure was applied directly to the chamber's inner surfaces. The rectangular end face of each finger and the four rectangular side faces of the central palm were fully constrained (*U_x_* = *U_y_* = *U_z_* = *U_Rx_* = *U_Ry_* = *U_Rz_* = 0), as they are fixed to the fixture base. TPE 85A filament with a material density of 1160 kg/m^3^ was modeled using a hyperelastic constitutive model. The Mooney‐Rivlin five‐parameter hyperelastic material constitutive model (see Text S3 for details) was adopted to capture the stress‐strain behavior of TPE 85A. Material parameters were derived from uniaxial tensile tests (Figure S5), yielding the constants *C*_10_ = −10.713 MPa, *C*_01_ = 16.189 MPa, *C*_11_ = −0.129 MPa, *C*_20_ = 0.012 MPa, *C*_02_ = 3.22 MPa, and *D*_1_ = 0 MPa. The resulting fitting curves based on the constitutive model show excellent agreement with the experimental stress–strain data.

### 4.4 Control System Design

The control system primarily consists of an Arduino microcontroller, relays, solenoid valves, and vacuum pumps. The SRSG's A1−A2 Fingers, B1−B2 Fingers, and central palm are individually controlled and driven through the following control procedure. The Arduino microcontroller commands a six‐channel relay (G2RL‐2, Shenzhen Biaokong Electric Technology Co., Ltd.) to actuate six solenoid valves (VK332V‐5G‐01, SMC Corp.) in a specified sequence. Three of these solenoid valves were connected to a vacuum pump (Fujiwara‐1550D, Taizhou Fujiwara Tools Co., Ltd.) capable of generating a negative pressure up to 98 kPa, enabling independent actuation of two pairs of diagonal fingers and the central palm. The remaining three solenoid valves were connected to the atmosphere to restore the gripper to the initial configuration. To ensure precise regulation of pneumatic pressure, a pressure sensor (SIN‐Y290, Hangzhou Liance Automation Technology Co., Ltd.) was integrated into the system to monitor the magnitude of pressure delivered from the pump to the gripper (see control system schematic diagram in Figure S18).

### 4.5 Performance Characterization of the Finger

To measure response time and tip trajectory of the finger, we performed experiments using a motion capture system (Prime41, OptiTrack Inc.) with an absolute accuracy of about 0.3 mm and a sampling frequency of 120 Hz. In each test, the initial internal pressure within the finger was equal to ambient atmospheric pressure. Upon application of −98 kPa, the real‐time position changes of the finger were recorded until the bending deformation reached a steady state. Similarly, to characterize the finger's bending angles, a camera (EOS 700D, Canon Inc.) was used to record its overall bending process under different negative pressures, and the corresponding bending angles were derived using an image analysis system. For cyclic durability characterization, Finger A was fixed on the test platform, when in the bent state, with its tip vertically aligned with the contact head of a force sensor (HANDPI, HP‐500). Negative pressure was applied to Finger A until its tip contacted the force sensor at −80 kPa, and then released to allow the finger to recover to its initial state. This vacuumizing and releasing process was repeated for 2000 cycles, and the output force and input pressure were recorded synchronously using the force sensor and a pressure sensor (DP‐101A, Hefei Lingguang Technology Co., Ltd.), respectively.

### 4.6 Output Force Characterization

The output forces of both a single finger and the complete SRSG were measured following a standardized testing protocol to evaluate the effects of the petals and finger arrangements on grasping performance. The SRSG was first mounted at the end of a six‐degree‐of‐freedom robotic arm (UR5, Universal Robots Inc.), which was held in either a horizontal or a vertical static pose to measure grasping forces and horizontal resistive forces. The gripper was then actuated under a negative pressure of 98 kPa to secure the target object. The force measurements were obtained using a load cell (HANDPI, HP‐500) fixed to a ball screw linear motion platform. The object was attached to the load cell via a fluorocarbon line, and then the platform pulled the object horizontally at a constant speed of 10 mm/s until complete detachment. The peak force data were recorded in real time from initial contact through full separation. Cylindrical and spherical objects with diameters of 30, 60, and 90 mm, as well as square plates with thicknesses of 4, 8, and 12 mm, were tested to comprehensively evaluate the output force performance of the SRSG when grasping different objects. For single finger characterization, the finger was clamped using aluminum profiles, and a similar procedure was performed using cylindrical objects of 15, 30, 45, and 60 mm in diameter. Each set of all the above experiments was repeated three times, and the results were reported as mean output forces.

## Author Contributions

**Qiping Xu**: conceptualization, data curation, formal analysis, funding acquisition, investigation, methodology, project administration, resources, supervision, validation, visualization, writing – original draft, writing – review and editing. **Bin Wang**: conceptualization, data curation, formal analysis, investigation, methodology, software, validation, visualization, writing – original draft, writing – review and editing. **Jinxin Chen**: formal analysis, investigation. **Zhengqiang Guo**: funding acquisition, validation. **Baisong Yang**: resources, investigation. **Chaoqian Chen**: resources, validation. **Jiancheng Cai**: resources, funding acquisition. **Chee‐Meng Chew**: investigation, methodology, validation, writing – original draft, writing – review and editing. **Shiju E**: funding acquisition, project administration, resources, supervision, writing – review and editing.

## Funding

This work was supported by National Natural Science Foundation of China (Grant No. 52476040), Key Project of Zhejiang Provincial Natural Science Foundation (Grant No. LZ24E070001), Zhejiang Provincial Natural Science Foundation (Grant No. LQ22A020003, LQN26E050014), and China Scholarship Council (Grant No. 202308330163).

## Conflicts of Interest

The authors declare no conflicts of interest.

## Supporting information




**Supporting File 1**: advs76403‐sup‐0001‐SuppMat.docx

**Supporting File 2**: advs76403‐sup‐0002‐MovieS1.mp4.

**Supporting File 3**: advs76403‐sup‐0003‐MovieS2.mp4.

**Supporting File 4**: advs76403‐sup‐0004‐MovieS3.mp4.

**Supporting File 5**: advs76403‐sup‐0005‐MovieS4.mp4.

**Supporting File 6**: advs76403‐sup‐0006‐MovieS5.mp4.

**Supporting File 7**: advs76403‐sup‐0007‐MovieS6.mp4.

**Supporting File 8**: advs76403‐sup‐0008‐MovieS7.mp4.

**Supporting File 9**: advs76403‐sup‐0009‐MovieS8.mp4.

**Supporting File 10**: advs76403‐sup‐0010‐MovieS9.mp4.

**Supporting File 11**: advs76403‐sup‐0011‐MovieS10.mp4.

**Supporting File 12**: advs76403‐sup‐0012‐MovieS11.mp4.

**Supporting File 13**: advs76403‐sup‐0013‐MovieS12.mp4.

**Supporting File 14**: advs76403‐sup‐0014‐MovieS13.mp4.

**Supporting File 15**: advs76403‐sup‐0015‐MovieS14.mp4.

## Data Availability

The data that support the findings of this study are available from the corresponding author upon reasonable request.
