# Supplementary material for: Biomimetic Self‐Reconfigurable Soft Gripper for Cross‐Scale, Multi‐Particle, and High‐Load Multifunctional Manipulation
Source: Adv Sci (Weinh). 2026 Jul 3;13(54):e76403. doi: 10.1002/advs.76403 (PMC13334592; doi:10.1002/advs.76403)
Supplement: Supplementary file 1 [file ADVS-13-e76403-s012.docx]

Supporting Information

Biomimetic self-reconfigurable soft gripper for cross-scale, multi-particle, and high-load multifunctional manipulation

*Qiping Xu*, Bin Wang, Jinxin Chen, Zhengqiang Guo, Baisong Yang, Chaoqian Chen, Jiancheng Cai, Chee-Meng Chew*, and Shiju E**

Supporting Information

**Self-reconfigurable soft gripper capable of switching finger arrangements and altering workspace**

Qiping Xu^1,2,3^*, Bin Wang^1,3^, Jinxin Chen^1^, Zhengqiang Guo^1^, Baisong Yang^1^, Chaoqian Chen^1^, Jiancheng Cai^1^, Chee-Meng Chew^2^* & Shiju E^1^*

^1^ Key Laboratory of Urban Rail Transit Intelligent Operation and Maintenance Technology & Equipment of Zhejiang Province, Department of Robotics Engineering, College of Engineering, Zhejiang Normal University, Jinhua, 321004, People’s Republic of China.

^2^ Department of Mechanical Engineering, College of Design and Engineering, National University of Singapore, 9 Engineering Drive 1, Singapore 117575, Singapore.

^3^ These authors contributed equally: Qiping Xu, Bin Wang.

*Corresponding authors: xuqiping@zjnu.edu.cn; mpeccm@nus.edu.sg; esx_2001@zjnu.cn

The file includes:

Supplementary Texts S1 to S5

Supplementary Figures S1 to S24

Supplementary Tables S1 to S8

Other Supplementary Materials for this manuscript include the following:

Movies S1 to S14

**Supplementary Texts**

**Text S1. Structural design of the petal modules**

The petal modules (hereafter referred to as petals) are categorized into two types, A and B, which are installed along the diagonals of the SRSG (Figure S1A). The overall configuration design of the petal modules is inspired by the morphology of natural rapeseed flower petals and their overlapping closure mechanism. Finite element analysis (FEA) was performed to simulate the fully closed state of the SRSG equipped with petals (Figure S2). Based on simulation results, the geometric shapes of the petals were optimized; that is, the front ends of petals A and B were designed with distinct shapes (trapezoidal and rectangular), and the overall width was appropriately increased to ensure that, upon closure, the petals overlap to form an almost enclosed structure. Compared with non-enclosing gripper designs, the petal-equipped SRSG creates a more stable enclosed space during closure, enabling robust enveloping grasping that effectively prevents small objects from slipping through gaps.

Ease of assembly and disassembly is also a critical design consideration. To achieve this, hollow trapezoidal sleeves were designed at the front end of each petal, enabling quick fingertip insertion for connection. The rear end features a dovetail joint, with its width *w_d_* slightly exceeding the finger width *w_f_*. This ensures that, when the dovetail joint at the rear of the petal connects with the dovetail groove at the base of the finger, it slightly protrudes from the finger sidewall. A gentle push on this protrusion allows for effortless disassembly, as shown in Figure S1B. This design not only enables rapid assembly and disassembly but also significantly simplifies the cumbersome assembly processes inherent in traditional designs, thereby improving operational flexibility while maintaining the structural strength of the petals.

Additionally, Petal A and Petal B are mounted at different heights on the SRSG (Figure S1B). This height difference avoids mutual interference of the petals during finger reconfiguration and facilitates the overlapping closure sequence, with Petal A overlapping Petal B. Furthermore, the fingers and the central palm are pneumatically actuated independently, as illustrated in the pneumatic control scheme in Figure S1C. Since the petals undergo passive deformation driven solely by finger bending at the joints, three trapezoidal grooves were incorporated on the petal surfaces in contact with the fingers (see Figure S1A and Table S1 for detailed dimensions). The petal thickness is *t*_1_ = 0.8 mm, while in each groove region the thickness is reduced to *t*_2_ = 0.4 mm. The groove positions are precisely aligned with the three movable joints of the fingers, enabling the petals to bend along grooves that function as creases. This design makes petal deformation predictable, significantly reduces the interference of petals on finger bending performance, and enhances the gripper’s enveloping grasping capability.

**Text S2. Kinematic modeling of the finger**

To simplify the motion analysis of the finger, we use rigid linkages with rotational joints to establish its kinematic model. Given the identical geometries and volumes of each chamber joint, and neglecting the gravitational effects of the finger’s self-weight, the rotation angle variation is assumed to be equal for each joint under unloaded conditions. With the base link of a single finger fixed, the tip trajectory of the finger can be determined by analyzing the motion of the three movable joints and links. The Denavit-Hartenberg (D-H) coordinate system is established based on the finger’s initial posture, as shown in Supplementary Figure S4. The base coordinate system (*x*_0_, *y*_0_, *z*_0_) is defined at the starting point of the fixed base link, with corresponding link coordinate systems at three movable joints. In each link coordinate system, the *z_i_* axis is oriented normal to the width direction of the finger, the *x_i_* axis extends along the length of the finger from the *z_i_* axis toward the *z_i+_*_1_ axis, and the *y_i_* axis is determined by the right-hand rule, perpendicular to the plane formed by the *x_i_* and *z_i_* axes. Here, *L_i_* denotes the length of the movable links, *L*_0_ denotes the length of the fixed base link, and *θ_i_* denotes the rotation angle between adjacent links. The specific parameter values are provided in Table S1.

The maximum bending angle *α* of the flexible joint with a U-shaped origami chamber is set to 65° (Figure 2E and Figure S4). Under negative pressure actuation, the joint bends until its sidewalls come into contact, after which further increases in negative pressure produce only negligible additional bending. All joints lie within the same plane and bend in the same direction, with each joint angle constrained to *θ_i_* ∈ [0°, 65°].

By establishing all link coordinate systems, the corresponding D-H parameters can be derived (Table S2), from which the position (*x*, *y*, *z*) of the end joint can be determined. For *i* > 1, the transformation matrix from coordinate system *i*-1 to coordinate system *i* is expressed as:

$${}_{i}^{i-1}T=\left[\begin{matrix} \cos\theta_{i} & -\sin\theta_{i} & 0 & a_{i}\cos\theta_{i} \\ \sin\theta_{i} & \cos\theta_{i} & 0 & a_{i}\sin\theta_{i} \\ 0 & 0 & 1 & 0 \\ 0 & 0 & 0 & 1 \end{matrix}\right]$$

By applying negative pressure, the U-shaped origami chamber bends along the predefined crease lines until reaching a preset angle. The bending angles and directions of all joints in the finger are identical, with *θ*_1_ = *θ*_2_ = *θ*_3_ = *θ*. The overall transformation matrix is given by:

$${}_{3}^{0}T={}_{1}^{0}T\cdot{}_{2}^{1}T\cdot{}_{3}^{2}T=\left[\begin{matrix} \cos(3\theta) & -\sin(3\theta) & 0 & X \\ \sin(3\theta) & \cos(3\theta) & 0 & Y \\ 0 & 0 & 1 & 0 \\ 0 & 0 & 0 & 1 \end{matrix}\right]$$

With the base coordinate system defined at the starting point of the fixed base link, the tip position coordinates (*X*, *Y*, *Z*) of the finger in the global coordinate system can be calculated as:

$$\begin{matrix} X=L_{0}+L_{1}\cos\theta+L_{2}\cos(2\theta)+L_{3}\cos(3\theta) \\ Y=L_{1}\sin\theta+L_{2}\sin(2\theta)+L_{3}\sin(3\theta) \\ Z=0 \end{matrix}$$

The established kinematic model enables accurate prediction of both finger deformation postures (Figures S6, S7) and tip trajectories of the fingers (Figure S13). Comparison with experimental results confirms the validity and accuracy of this kinematic model.

**Text S3. Determination of material parameters in the hyperelastic constitutive model**

The SRSG was fabricated from TPE 85A. To determine the material parameters of TPE 85A, uniaxial tensile tests were performed to obtain the experimental data. Following the ASTM D638 standard, five dumbbell-shaped specimens were monolithically printed via fused deposition modeling (FDM) using TPE 85A. After fabrication, the geometric dimensions of each specimen were measured using a micrometer to calculate their cross-sectional area (Figure S5A). Subsequently, uniaxial tensile tests were conducted using a universal testing machine (Z1.0, Zwick/Roell) equipped with a 1 kN load cell and a crosshead displacement sensor (Figure S5B). Each specimen was tested at a strain rate of 50 mm/min, with load and displacement data recorded at a sampling frequency of 50 Hz. The nominal stress and nominal strain were calculated from the measured data, and their mean curves were obtained from the five specimens (Figure S5C). The experimental data were input into the commercial finite element software ABAQUS and fitted based on four different hyperelastic constitutive models to determine the coefficients (i.e., material parameters) of their strain energy density functions. The expressions of these models are given below:

Neo-Hookean:

$$W = C_{10}\text{(}I_{1}-\text{3)}$$

Ogden (N=1):

$$W=\sum_{i=1}^{N} \frac{2\mu_{i}}{\alpha_{i}^{2}}(\lambda_{1}^{\alpha_{i}}+\lambda_{2}^{\alpha_{i}}+\lambda_{3}^{\alpha_{i}}-3)$$

Yeoh:

$$W=C_{10}(I_{1}-3)+C_{20}(I_{1}-3)^{2}+C_{30}(I_{1}-3)^{3}$$

Mooney-Rivlin five:

$$\begin{matrix} W=C_{10}\left(I_{1}-3\right)+C_{01}\left(I_{2}-3\right)+C_{20}\left(I_{1}-3\right)^{2} \\ \text{ }+C_{11}\left(I_{1}-3\right)\left(I_{2}-3\right)+C_{02}\left(I_{2}-3\right)^{2}+\frac{1}{D_{1}}(J-1)^{2} \end{matrix}$$

Uniaxial tensile test data were fitted using several hyperelastic constitutive models to evaluate their predictive accuracy. The results indicate that the five-parameter Mooney-Rivlin model provides the best agreement with the experimental data (Figure S5D) and was therefore adopted as the constitutive model for TPE 85A. Here, *I*_1_ and *I*_2_ denote the first and second strain invariants, respectively, characterizing the degree of deformation, and *J* represents the ratio of post-deformation volume to pre-deformation volume (*J* = 1 for incompressible materials). *C*_10_, *C*_01_, *C*_11_, *C*_20_, *C*_02_, and *D*_1_ are the material parameters of the hyperelastic constitutive model. For the five-parameter Mooney-Rivlin model, these parameters were determined as: *C*_10_ = –10.713 MPa, *C*_01_ = 16.189 MPa, *C*_11_ = –0.129 MPa, *C*_20_ = 0.012 MPa, *C*_02_ = 3.22 MPa, and *D*_1_ = 0 MPa.

**Text S4. Output torque modeling of the origami chamber joint**

The motion of the origami chamber joints on the fingers and palm is approximated as rotational motion about an equivalent rotation center. To simplify theoretical calculation, the thickness of the chamber walls is neglected. Taking a typical single U-shaped origami chamber as the research object (Figure S8A), its total volume *V_U_* is divided into two components: the triangular prism volume *V_S_* and the central origami volume *V_P_*, which can be further approximated as two identical quadrangular pyramids, each with a volume of $\frac{\text{V}\text{P}}{\text{2}}$. When negative pressure is applied, the central origami chamber surface folds inward along the creases until the adjacent chamber walls overlap with each other (Figure S8B). It is assumed that the triangular prism volume *V_S_* remains constant, while the quadrangular pyramid volume *V_P_* varies dynamically with the joint rotation. The slant edge length of the side triangle of the triangular prism is *l*, and the included angle between the two slant edges is *α* (maximum bending angle of the finger joints), which decreases to *α* – *θ* after deformation, where *θ* is the real-time rotation angle of the joint. The distance between the rotation center and the crease center is *h_c_*, and the perpendicular height of the side triangle of the triangular prism is *h_t_*. Based on the theoretical geometric relationship, the following formulas can be derived:

$$V_{S}=\frac{1}{2}w_{f}lh_{p}$$

$$V_{P}=\frac{2}{3}w_{f}lh_{c}\sin\left(\frac{\alpha-\theta}{2}\right)$$

By combining Eqs. (S8) and (S9), the volume of the U-shaped origami chamber is obtained as:

$$V_{U}=V_{S}+V_{P}=\frac{1}{2}w_{f}lh_{p}+\frac{2}{3}w_{f}lh_{c}\sin\left(\frac{\alpha-\theta}{2}\right)$$

The deformation of the origami chamber joint under negative pressure was treated as a quasi‑static rotation process. In this process, the negative-pressure-induced input work within the chamber, *dW_in_*, balances the output work produced by chamber joint rotation, *dW_out_*:

$$dW_{in}\text{ }=\text{ }dW_{out}$$

$$PdV_{U}=M_{i}d\theta$$

Here, *P* is the negative pressure acting on the internal chamber, and *dV_U_* refers to the internal volume variation of the origami chamber. *M_i_* denotes the output torque generated by a single origami chamber, and *dθ* represents the rotation angle variation of the chamber joint. In this simplified analytical model, the elastic deformation of the TPE material, the self-weight of the chamber joint, and other dissipative energies are neglected. By combining Eqs. (S8) to (S12), the output torque *M_i_* of a single chamber joint can be expressed as:

$$M_{i}=P\frac{dV}{d\theta}=-\frac{1}{3}Pw_{f}lh_{c}\cos\left(\frac{\alpha-\theta}{2}\right)$$

For multiple identical chamber joints, the total output torque *M* is:

$$M=n\cdot M_{i}=-\frac{1}{3}nPw_{f}lh_{c}\cos\left(\frac{\alpha-\theta}{2}\right)$$

where *n* is the number of chambers.

**Text S5. Calculation of the enclosed space volume of the SRSG**

The enclosed volume *V*_Ⅰ_ of Gripper I can be approximated as *V_M-_*_Ⅰ_ – *V_G-_*_Ⅰ_, where *V_M-_*_Ⅰ_ represents the volume of a double square frustum with four corner regions removed (Figure S21A), and *V_G-_*_Ⅰ_ represents the volume of the fully closed Gripper I. The relationship is expressed as:

$$V_{\text{I}}=V_{M-\text{I}}-V_{G-\text{I}}=4V_{H}+V_{C}-V_{G-\text{I}}$$

where *V_H_* denotes the volume of the heptahedron, and *V_C_* denotes the volume of the cuboid, which are calculated as:

$$V_{H}=\frac{w_{f}\left[(b_{1}+b_{2}-2 w_{f}) d_{1}+(b_{1}+b_{3}-2 w_{f}) d_{2}\right]}{4}$$

$$V_{C}=w_{f}^{2}(d_{1}+d_{2})$$

Thus,

$$V_{\text{I}}=w_{f}\left[(b_{1}+b_{2}-2w_{f})d_{1}+(b_{1}+b_{3}-2w_{f})d_{2}+w_{f}(d_{1}+d_{2})\right]-V_{G-\text{I}}$$

The enclosed volume *V*_Ⅱ_ of Gripper Ⅱ can be approximated as *V_M-_*_Ⅱ_ – *V_G-_*_Ⅱ_, where *V_M-_*_Ⅱ_ represents the volume of a complete double square frustum (Figure S21B), and *V_G-_*_Ⅱ_ represents the volume of the fully closed Gripper Ⅱ. Since the petals are extremely thin, their volume can be neglected, and thus *V_G-_*_Ⅰ_ and *V_G-_*_Ⅱ_ can be reasonably approximated as equal (*V_G-_*_Ⅰ_ = *V_G-_*_Ⅱ_). The relationship is expressed as:

$$V_{\text{II}}=V_{M-\text{II}}-V_{G-\text{I}}=V_{T1}+V_{T2}-V_{G-\text{I}}$$

where *V_T_*_1_ and *V_T_*_2_ denote the volumes of the upper and lower square frusta, respectively, which are calculated as:

$$V_{T1}=\frac{d_{1}\left(b_{1}^{2}+b_{2}^{2}+b_{1}b_{2}\right)}{3}$$

$$V_{T2}=\frac{d_{2}\left(b_{1}^{2}+b_{3}^{2}+b_{1}b_{3}\right)}{3}$$

Thus,

$$V_{\text{II}}=\frac{d_{1}(b_{1}^{2}+b_{2}^{2}+b_{1}b_{2})+d_{2}(b_{1}^{2}+b_{3}^{2}+b_{1}b_{3})}{3}-V_{G-\text{I}}$$

The side profiles of Gripper I and Gripper II in the fully closed state are approximated by a combination of two trapezoidal cross-sections, corresponding to the upper (*V_T_*_1_) and lower (*V_T_*_2_) square frustums, respectively. Here, *d*_1_ and *d*_2_ denote the heights of the trapezoidal sections for *V_T_*_1_ and *V_T_*_2_, respectively, while *b*_1_ and *b*_2_ denote the shorter sides of the two trapezoidal sections. *b*_3_ is the common longer side shared by both trapezoidal sections (highlighted in red in Figure S21A, B). The geometric parameters used are: *b*_1_ = 117 mm, *b*_2_ = 90 mm, *b*_3_ = 79 mm, *d*_1_ = 23.38 mm, *d*_2_ = 32.91 mm.

To calculate the total volume of the gripper in its closed state, all internal chambers and pneumatic channels are treated as solid regions. Finite element simulations show that, under negative-pressure actuation, the U-shaped origami chambers in each finger joint collapse until the opposite sidewalls come into contact (Figure 2F and Figure S9), with *V_P_* denoting the volume reduction of a single collapsed chamber (Figure S21C). Accordingly, the closed-state volume *V_G-_*_Ⅰ_ of the gripper is defined as the undeformed gripper volume *V_G_* minus the total collapsed volume 12*V_P_* (three chamber joints per finger, four fingers in total), and this resulting relationship is expressed as:

$$V_{G-\text{I}}=V_{G}-12V_{P}$$

Therefore, the enclosed volumes of Grippers I and II are determined by Eq. (S18) and Eq. (S22), respectively.

**Figure S1. Schematic of the SRSG and petal modules, illustrating structural and assembly details.** (A) Installation positions and detailed structural design of Petals A and B in the SRSG. (B) Mounting heights of Petals A and B on the SRSG are denoted as *h*_1_ and *h*_2_, respectively. (C) Pneumatic circuit schematic of the SRSG. The fingers and central palm are actuated independently by negative pressure, where *P*₀ and *Pᵢ* denote the actuation pressures of the palm and fingers, respectively.

**Figure S2. Finite element analysis (FEA) of the SRSG with petal modules in the fully closed state.**

**Figure S3. Monolithic fabrication process via FDM 3D printing.**

**Figure S4. Schematic of the finger structure.**

**Figure S5. Characterization of TPE 85A material.** (A) Specimen fabricated by 3D printing. (B) Tensile tests of five samples using the Zwick testing machine. (C) Stress-strain curve of TPE 85A. (D) Fitting of tensile data using different hyperelastic constitutive models.

**Figure S6. Finger bending deformation under the negative pressure of 30 kPa.** Comparison of results of four fingers from (A) experimental measurement, (B) FEA, and (C) the proposed kinematic model.

**Figure S7. Finger bending deformation under the negative pressure of 60 kPa.** Comparison of results of four fingers from (A) experimental measurement, (B) FEA, and (C) the proposed kinematic model.

**Figure S8.** **Schematic illustration of volume variation during folding-induced rotational deformation of the origami chamber joint.** **(A)** Decomposition of the total volume *V_U_* of a single U-shaped origami chamber into the triangular prism volume *V_S_* and the central origami chamber volume *V_P_*, where *V_U_* = *V_S_* + *V_P_*. The central origami chamber volume is further approximated as two identical quadrangular pyramids, each with a volume of $\frac{\text{V}_{\text{P}}}{\text{2}}$. **(B)** Side view of the rotational deformation. The chamber is assumed to rotate about the rotation center, with the angle decreasing from *α* to *α* – *θ* during inward folding.

**Figure S9. FEA of folding deformation of the U-shaped origami chamber, shown in cross-sectional view.**

**Figure S10. Two contact modes of fin-like fingertips.** The structure adaptively switches between pinching and conforming contact based on object geometries.

**Figure S11. FEA of fingertip-object contact at two different locations.** Comparison of contact between fin-like and solid fingertips: (A, B) midsection contact and (C, D) tip contact.

**Figure S12. Temporal profiles of Finger A and the central palm during a complete actuation cycle.** Bending angle versus time for Finger A and the central palm under one bending and recovery cycle at −98 kPa. Finger A reaches the stable bending state in approximately 0.211 s and returns to the completely unactuated state in about 0.33 s, whereas the corresponding bending and recovery times for the central palm are roughly 0.13 s and 0.25 s, respectively.

**Figure S13. Comparison of the analytical model and experimentally measured tip trajectories of the four fingers under different tip payloads.** (A) Representative experimental results of Finger A in the initial and actuated states under three tip payload conditions (0, 10, and 50 g). The masses were suspended at the fingertip to evaluate the influence of the external payload on finger deformation. (B) Tip trajectory of Finger A. (C) Tip trajectory of Finger B. (D) Tip trajectory of Finger C. (E) Tip trajectory of Finger D.

**Figure S14. Experimental setup for measuring the grasping force of an individual finger.**

**Figure S15. Experimental setup and procedure for the cyclic durability test of Finger A.**

**Figure S16. Experimental setups for output force characterization of the SRSG.** (A) Measuring grasping forces. (B) Measuring horizontal resistive forces.

**Figure S17. Comparison of output forces when grasping different objects using two-finger and four-finger grippers.** (A, B) Maximum grasping forces when manipulating plates and cylinders of varying sizes. (C, D) Maximum horizontal resistive force when grasping plates and cylinders of different sizes. All experiments were performed using grippers without petals.

**Figure S18. Control system architecture of the SRSG, comprising an Arduino microcontroller, a six-channel relay, vacuum pumps, and six solenoid valves.**

**Figure S19. The SRSG rotates bulbs with diameters of 70, 95, and 135 mm.**

**Figure S20. Analysis of contact area and fingertip position during bulb rotation.** (A) Contact state before rotation. (B) Contact state after rotation. S1–S4 denote the gap areas between the fingers and the bulb surface at different stages of the rotation process.

**Figure S21. Closed-state volume analysis of the SRSG.** (A) Closed-state volume of Gripper I, approximated as a double square frustum with the four corner regions removed. (B) Closed-state volume of Gripper II, approximated as a double square frustum comprising two stacked square frusta sharing a common base. (C) Equivalent solid model, including the undeformed gripper volume *V_G_* and the collapsed volume *V_P_* of a single chamber joint.

**Figure S22. Continuous grasping of various fruits and vegetables.** (A) Representative grasping sequence, exemplified by grasping a chili pepper. (B) Self-reconfiguration process of the SRSG. (C) Initial and target positions of the objects. (D−I) The SRSG sequentially grasps different items and places them in order on the tray.

**Figure S23. Grasping stability tests.** (A) The grasped object is translated along a rectangular path, rotated about the UR5 robotic arm’s end-joint axis, and subsequently returned to its initial position. (B) The grasped object is rotated 180° clockwise and then returned to its initial position. (C) The grasped object is rotated 180° counterclockwise and then returned to its initial position. In all tests, the object is a plastic bucket containing a football.

**Figure S24. The initial position and grasping sequence of the objects.**

**Table S1. Description of specific parameters.**

| **Description** | **Parameters** | **Value** |
| --- | --- | --- |
| Total height of the Finger | *H* | 18 mm |
| Total width of the Finger | *w_f_* | 18 mm |
| Distance between the rotation center and the crease center | *h_c_* | 11 mm |
| Slant edge length of the side triangle of the triangular prism | *l* | 20 mm |
| Perpendicular height of the side triangle of the triangular prism | *h_t_* | 8 mm |
| Finger Base Solid Segment | *L*_0_ | 27 mm |
| First movable Solid Segment of Finger | *L*_1_ | 27 mm |
| Second movable Solid Segment of Finger | *L*_2_ | 38 mm |
| Third movable Solid Segment of Finger | *L*_3_ | 42 mm |
| The wall thickness of the Finger chamber | *T* | 0.8 mm |
| Maximum bending angle of the finger joints | *α* | 65° |
| The angle of inclination of the fingertip | *φ* | 30° |
| The angle between the fingertip rib plate and the base surface | *β* | 120° |
| Total length of the petal module | *L_P_* | 125 mm |
| The width of the dovetail joint on the petal component | *w_d_* | 22 mm |
| Thickness of the petal module | *t*_1_ | 0.8 mm |
| The thickness of the trapezoidal groove in the petal module | *t*_2_ | 0.4 mm |
| Mounting height of petal module A | *h*_1_ | 2.4 mm |
| Mounting height of petal module B | *h*_2_ | 0.4 mm |

The above parameters are shown in Figures S1, S4, and S8.

**Table S2. D-H parameters for the equivalent revolute joints.**

| **Link *i*** | ***a_i_*** | ***α_i_*** | ***d_i_*** | ***θ_i_*** |
| --- | --- | --- | --- | --- |
| 1 | *L*_1_ | 0 | 0 | *θ*_1_ |
| 2 | *L*_2_ | 0 | 0 | *θ*_2_ |
| 3 | *L*_3_ | 0 | 0 | *θ*_3_ |

**Table S3. Comparison of performance metrics of representative rotary actuators.**

| **References** | **Actuation mode** | **Torque (N∙mm)** | **Response time (s)** | **Continuous bidirectional rotation** |
| --- | --- | --- | --- | --- |
| This work | Negative pressure | ~121 | ~0.13 | Yes |
| [42] | Positive pressure | ~96 | >0.7 | Yes |
| [43] | Positive pressure | ~82 | >0.7 | Yes |
| [45] | Negative pressure | ~63 | ~0.47 | No |
| [46] | Negative pressure | ~24 | ~0.7 | No |
| [47] | Negative pressure | ~95 | ~0.3 | No |

**Table S4. List of the grasped objects.**

| **Categories** | **Weight**  **(g)** | **Grasping Size (mm)** | **Grasping Modes** |
| --- | --- | --- | --- |
| Human hair | 0.0004 | 0.07 | Parallel |
| Fluorocarbon line | 0.006 | 0.2 | Parallel |
| Rice grain | 0.05 | 1 | Enveloping |
| M3 screw | 2 | 3 | Parallel |
| Loquat stem | 18 | 4 | Parallel |
| Soybean | 0.2 | 5 | Enveloping |
| Foam ball | 0.1 | 15 | Enveloping |
| Longan | 11 | 23 | Diagonal |
| Bayberry | 13 | 26 | Diagonal |
| Lychee | 29 | 38 | Diagonal |
| Banana | 165 | 42 | Parallel |
| Sea cucumber | 78 | 43 | Parallel |
| Orange | 48 | 45 | Diagonal |
| Chili pepper | 99 | 53 | Parallel |
| Crayfish | 35 | 56 | Enveloping |
| Crab | 50 | 67 | Enveloping |
| Apple | 168 | 71 | Diagonal |
| Conch | 82 | 73 | Parallel |
| Mango | 187 | 77 | Diagonal |
| Durian pulp | 217 | 83 | Parallel |
| A bunch of tangerines | 184 | 88 | Enveloping |
| A bracket loaded with weights | 5600 | 98 | Diagonal |
| Single-sided tape | 145 | 100 | Diagonal |
| Scallop | 72 | 101 | Diagonal |
| Sea urchin | 129 | 108 | Diagonal |
| Porcelain vase | 1488 | 110 | Diagonal |
| A bunch of cherry tomatoes | 232 | 110 | Enveloping |
| A pack of tissues | 135 | 130 | Parallel |
| Porcelain bowl | 313 | 145 | Diagonal |
| A bunch of grapes | 378 | 150 | Enveloping |
| Football | 227 | 170 | Diagonal |
| Porcelain plate | 464 | 180 | Diagonal |
| Plastic bucket | 312 | 230 | Diagonal |
| Square plate | 166 | 270 | Diagonal |

**Table S5. Parameters and output characteristics of several typical grippers.**

| **Gripper type** | **Size**  **(mm)** | **Weight**  **(g)** | **Grasping Force**  **(N)** | **Grasping range ratio** |
| --- | --- | --- | --- | --- |
| Finger [12] | 80×20×20 | N/A | 36 | 6 |
| Finger [18] | 176×30×30 | N/A | 2 | 8 |
| Finger [19] | N/A | 750 | 8 | 3 |
| Finger [20] | 140×20×18 | N/A | 16 | 13 |
| Finger [22] | N/A | N/A | 6.5 | 120 |
| Finger [39] | 100×60×170 | 380 | 21 | 360 |
| Suction [13] | 28.08×20.38×45 | N/A | 5 | 3 |
| Suction [31] | 73×73×20 | 170 | 25 | 8 |
| Suction [32] | 240×240×20 | N/A | 10 | 20 |
| Suction [49] | 60×60×30 | N/A | 21 | 32 |
| Continuum [14] | N/A | N/A | 60 | 6 |
| Continuum [50] | 84.45×32.47×32.47 | N/A | 3 | 3 |
| Continuum [51] | 507×88×88 | N/A | 40 | 120 |
| Foldable [15] | N/A | N/A | 32 | 4 |
| Foldable [52] | 90×90×50 | N/A | 31 | 6 |
| Foldable [53] | N/A | N/A | 1.06 | 50 |
| Foldable [54] | N/A | N/A | 11 | 6 |
| Wrapping [16] | 40×40×65 | N/A | 20 | 13 |
| Wrapping [55] | N/A | N/A | 6 | 4 |
| Wrapping [56] | 55×55×60 | 101.3 | 28 | 5 |

**Table S6. Success rate of multiple grasping (5 tests for each case).**

|  | | | | |
| --- | --- | --- | --- | --- |
|  |  |  |  |  |
| Objects | Gripper Ⅰ | Gripper Ⅱ | Gripper Ⅲ | Gripper Ⅳ |
| 1. Fluorocarbon line | 0/5 | 0/5 | 4/5 | 4/5 |
| 2. M3 screw | 1/5 | 1/5 | 5/5 | 5/5 |
| 3. Soccer ball | 5/5 | 5/5 | 2/5 | 2/5 |
| 4. Plastic bucket | 5/5 | 5/5 | 0/5 | 0/5 |
| 5. Bayberry | 5/5 | 5/5 | 5/5 | 5/5 |
| 6. Apple | 5/5 | 5/5 | 3/5 | 3/5 |
| 7. Chili pepper | 4/5 | 4/5 | 5/5 | 5/5 |
| 8. Banana | 3/5 | 3/5 | 5/5 | 5/5 |
| Average ± SD | 0.7 ± 0.37 | 0.7 ± 0.37 | 0.73 ± 0.36 | 0.73 ± 0.36 |

**Table S7. Comparison of performance metrics between SRSG and tendon-driven grippers.**

| **References** | **Actuation**  **mode** | **Response time (s)** | **Grasping force (N)** | **Self-weight (g)** | **Payload-to-weight ratio** | **Structure composition** | **Control complexity** |
| --- | --- | --- | --- | --- | --- | --- | --- |
| This work | Vacuum | ~0.211 | 56 | 53 | ~106 | Monolithic | Low |
| [12] | Tendon | ~0.6 | 36 | N/A | N/A | Modular | High |
| [14] | Tendon | ~1 | 60 | >100 | <60 | Modular | High |
| [57] | Tendon | ~5 | 17 | >58 | <29.3 | Modular | High |
| [58] | Tendon | ~2.2 | 72 | ~5646 | ~1.3 | Modular | High |
| [59] | Tendon | ~1.1 | 25 | N/A | N/A | Modular | High |
| [60] | Tendon | ~0.75 | 31.25 | N/A | N/A | Modular | High |
| [61] | Tendon | ~2 | 31.06 | N/A | N/A | Modular | High |

**Table S8. Printing parameters of the SRSG.**

| **Parameter** | **Value** |
| --- | --- |
| Layer Height | 0.16 mm |
| Top Wall Thickness | 0.8 mm |
| Vertical Wall Thickness | 1.2 mm |
| Extrusion Width | 0.4 mm |
| Infill Density | 40% |
| Infill Flow Rate | 100% |
| Infill Pattern Type | Rectilinear |
| Infill Extrusion Width | 100% |
| Maximum Volume Velocity | 2.8 mm^3^/s |
| Number of Wall Layers | 2 |
| Bottom Solid Fill Layers | 4 |
| Top Solid Fill Layers | 6 |
| Bottom Solid Fill Flow Rate | 100% |
| Top Solid Fill Flow Rate | 100% |
| Bottom Solid Fill Pattern Type | Lines |
| Top Solid Fill Pattern Type | Lines |
| Bottom Solid Fill Extrusion Width Percentage | 100% |
| Top Solid Fill Extrusion Width Percentage | 100% |
| Generate Support | None |
| Brim Type | Inner Brim and Outer Brim |
| Brim Width | 9 mm |
| Brim Gap | 0.1 mm |
| Print Panel Type | Textured PEI panel |
| Heated Bed Temperature | 45 °C |
| Extruder Temperature | 225 °C |
| Default Printing Speed | 80 mm/s |
| First Layer Speed | 50 mm/s |
| Outer Wall Speed | 80 mm/s |
| Inner Layer Speed | 80 mm/s |
| Infill Speed | 80 mm/s |
| Jointing Speed | 80 mm/s |
| Bridging Speed | 50 mm/s |
